# Cardiac structure and function 1.5 years after COVID-19: results from the EPILOC study

**DOI:** 10.1007/s15010-025-02481-4

**Published:** 2025-02-24

**Authors:** Jana Schellenberg, Lynn Matits, Daniel A. Bizjak, Peter Deibert, Birgit Friedmann-Bette, Siri Göpel, Uta Merle, Andreas Niess, Norbert Frey, Oliver Morath, Gunnar Erz, Raphael S. Peter, Alexandra Nieters, Dietrich Rothenbacher, Winfried V. Kern, Jürgen M. Steinacker

**Affiliations:** 1 Sports and Rehabilitation Medicine, University Hospital Ulm, Leimgrubenweg 14, 89075 Ulm, Germany; 2 Clinical & Biological Psychology, Institute of Psychology and Education, Ulm University, Ulm, Germany; 3 Institute for Exercise and Occupational Medicine, Faculty of Medicine, Albert-Ludwigs-University of Freiburg, Freiburg, Germany; 4 Department of Internal Medicine VII, Sports Medicine, University Medical Hospital, Heidelberg, Germany; 5 Department of Internal Medicine I, University Hospital of Tuebingen, Tuebingen, Germany; 6 Department of Internal Medicine IV, Medical Centre, Faculty of Medicine, University of Heidelberg, Heidelberg, Germany; 7 Department of Sports Medicine, University Hospital of Tuebingen, Tuebingen, Germany; 8 Department of Cardiology, Angiology, and Pneumology, Heidelberg University Hospital, German Centre for Cardiovascular Research DZHK, Heidelberg, Germany; 9 Institute of Epidemiology and Medical Biometry, Ulm University, Ulm, Germany; 10 Institute for Immunodeficiency, Medical Centre, Faculty of Medicine, Albert-Ludwigs-University, Freiburg, Germany; 11 Division of Infectious Diseases, Department of Medicine II, Medical Centre, Faculty of Medicine, Albert- Ludwigs-University of Freiburg, Freiburg, Germany; 12 Institute for Rehabilitation Medicine Research, Ulm University, University Hospital Ulm, Sports and Rehabilitation Medicine, Bad Buchau, Germany

**Keywords:** Myocardial inflammation, SARS-CoV-2, Deformation imaging, Post-COVID syndrome, Long-term cardiac sequalae

## Abstract

**Purpose:**

Impaired left and right ventricular (LV/RV) function during acute SARS-CoV-2 infection has been predominantly reported in hospitalized patients, but long-term cardiac sequelae in large, well-characterized cohorts remain inconclusive. This study evaluated cardiac structure and function in individuals with post-Coronavirus disease (COVID) syndrome (PCS) compared to recovered controls (CON), focusing on associations with cardiopulmonary symptoms and rapid physical exhaustion (RPE).

**Methods:**

This multicenter, population-based study included 1154 participants (679 PCS, 475 age- and sex matched CON; mean age 49 ± 12 years; 760 women) 1.5 years post-infection. Transthoracic echocardiography assessed LV global longitudinal strain (GLS), RV GLS and RV free wall strain (FWS), and other measures. Cardiopulmonary exercise testing (CPET) measured maximum respiratory oxygen uptake (VO_2_max) as a marker of cardiopulmonary fitness.

**Results:**

PCS participants exhibited significantly lower LV GLS (-20.25% [-21.28 – -19.22] vs. -20.73% [-21.74 – -19.72], *p* = 0.003), reduced diastolic function (E/A 1.16 [1.04–1.27] vs. 1.21 [1.1–1.32], *p* = 0.022) and decreased TAPSE (24.45 mm [22.14–26.77] vs. 25.05 mm [22.78–27.32], *p* = 0.022) compared to CON, even after adjusting for confounders. RV strain values were similar between groups. LV GLS correlated inversely with VO_2_max (*p* = 0.004) and positively with RPE (*p* = 0.050), though no associations were observed with other cardiopulmonary symptoms.

**Conclusions:**

This study demonstrates subtle yet consistent reductions in LV function, specifically LV GLS and diastolic function, and exercise capacity in PCS compared to CON. While these changes are within reference ranges, their potential impact on clinical outcomes warrants further investigation. These findings highlight the need for cardiac assessments and long-term follow-up in symptomatic PCS patients.

**Supplementary Information:**

The online version contains supplementary material available at 10.1007/s15010-025-02481-4.

## Introduction

The Severe Acute Respiratory Syndrome-Coronavirus-2 (SARS-CoV-2) pandemic, initiated in 2019, caused significant global morbidity and mortality [1]. While SARS-CoV-2 primarily targets the respiratory system, myocardial involvement is linked to increased mortality [2, 3]. Reports on cardiac structural and functional alterations due to COVID-19 vary widely, with prevalence ranging from 3 to 78%, influenced by factors such as infection severity, intensive care requirements, age, pre-existing cardiovascular conditions, imaging techniques, and timing of evaluation [3, 4, 5]. Acute SARS-CoV-2 infection has been associated with right ventricular (RV) dysfunction [6, 7, 8] and, to a lesser extent, left ventricular (LV) impairment [9] in hospitalized patients. However, the long-term cardiac effects of SARS-CoV-2 infection remain inadequately understood, particularly in patients with persistent post-COVID symptoms.

Current evidence highlights the values of left ventricular global longitudinal strain (LV GLS) assessed by speckle-tracking echocardiography (STE) as a sensitive marker of subclinical LV dysfunction, which may not be detected by LV ejection fraction (EF) alone [10]. Impaired LV GLS and RV GLS during acute infection have been associated with increased mortality [6] and have been observed in both hospitalized and recovered individuals, irrespective of fitness level [11, 12, 13, 14, 15, 16]. RV remodelling has also been reported during acute infection and at three-months follow-ups [17, 18]. While some studies indicate persistent reductions in LV GLS and RV GLS after infection [19, 20], others report improvement over time [13, 19, 21, 22, 23]. These discrepancies underscore the need for robust long-term observations. Furthermore, the cardiac manifestations of post-COVID syndrome (PCS) remain underexplored, especially regarding the extent to which patients with PCS presenting at long-COVID clinics experience subclinical or overt cardiac dysfunction. Despite growing evidence of chronic fatigue (CF), rapid physical exhaustion (RPE), and cardiopulmonary symptoms in PCS [14, 24], the relationship between these symptoms and echocardiographic findings remain unclear. This multicenter cohort study aims to address the knowledge gap by evaluating long-term cardiac structure and function in individuals with post-COVID syndrome (PCS) compared to recovered controls (CON). It further explores associations between echocardiographic findings, cardiopulmonary symptoms, CF or rapid physical exhaustion (RPE), and maximum oxygen uptake, providing critical insights into the long-term cardiac effects of SARS-CoV-2 infection.

## Methods

### Study population and study design

**Fig. 1:**
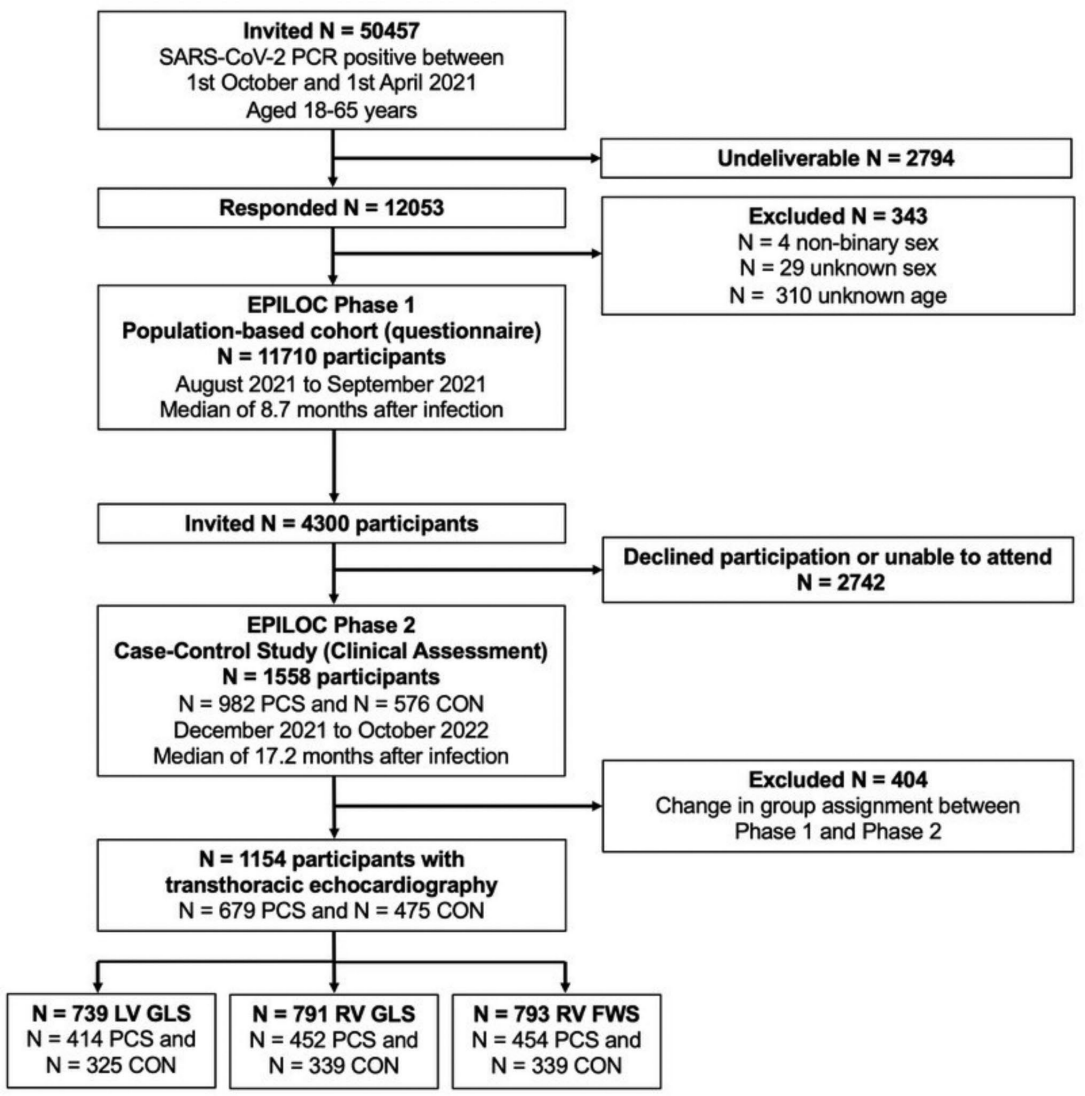
Flow Chart about EPILOC Phase 1 and Phase 2. The flowchart outlines the recruitment of PCS cases and controls matched by age and sex categories in EPILOC Phases 1 and 2 (adapted to Peter et al. 2024 [25]). Abbreviations: TTE = transthoracic echocardiography, LV GLS = left ventricular global longitudinal strain, RV = right ventricle, RV FWS = right ventricle free wall strain

The EPILOC (Epidemiology of Long COVID) Phase 2 study is a multicenter, non-interventional, population-based study conducted in southwest Germany. From the 50,457 invited subjects, 11,710 participants were enrolled six to twelve months after SARS-CoV-2 infection in the questionnaire-based Phase 1 [24]. Eligible participants were aged 18 to 65 years with a confirmed positive SARS-CoV-2 PCR test between October 1, 2020, and April 1, 2021. PCR testing was the primary method for participant inclusion and diagnostic confirmation due to its higher sensitivity and accuracy in detecting infection, especially in asymptomatic individuals. The participants were recruited through the regional health authorities, ensuring a broad and representative sample of the general population affected by COVID-19. PCS cases were defined as individuals experiencing one or more new symptoms (out of a catalogue of complaints more or less typical of Long COVID) persisting six to 12 months after acute infection, associated with at least moderate impairment of daily life and a general health status or work ability ≤ 80% compared to pre- infection levels. Symptom-free participants with a health status or work ability of 100% were included as recovered control group (CON). For Phase 2, 4300 participants (PCS cases and controls, frequency-matched by age and sex) were invited for clinical assessment, conducted between December 2021 to October 2022 (median time since acute infection, 17.2 months, ranging from 9.2 to 24.4 months) (Fig. 1) (for detailed description see [25]). The study population included individuals with different variants of SARS-CoV-2, primarily Alpha and early Delta variants, which were circulating during the study period. While we did not specifically stratify participants based on variant, we acknowledge that the diverse variants could have influenced the severity of infection and the subsequent recovery, including cardiovascular outcomes. Exclusion criteria were: acute or chronic illness that precluded a planned physical examination, acute SARS-CoV-2 infection, and withdrawal from study participation. The study was conducted in accordance with the Declaration of Helsinki and approved by the ethics committee of the Universities of Freiburg (EK 21-1484_1 and 490/21), Heidelberg (S-846/2021), Tübingen (845/2021BO2) and Ulm (337/21) and registered in the German Register of Clinical Studies database (DRKS00027012). Informed consent was obtained through signed consent forms at the study visit.

### Transthoracic echocardiography

Transthoracic echocardiography (TTE) was performed by experienced operators at each study center following international guideline recommendations [26]. The ultrasound devices used at the study centers are listed in the appendix (Supplemental Table 1). Measurements included LV EF in the apical four-chamber (LV EF A4C) and two-chamber views (LV EF A2C), indexed LV end-diastolic (LV EDVi A4C/A2C) and end-systolic volumes (LV ESVi A4C/A2C), left atrial (LA) and right atrial (RA) size, and diastolic function characterized by the E/A ratio and E/E´lateral and medial ratios [27]. RV dimensions and function were assessed, including basal and midventricular diameters, RV length, and tricuspid annular plane systolic excursion (TAPSE). All analyses were performed by the same investigator in each center. LV GLS and RV GLS/FWS were determined using TomTec postprocessing software (AutoStrain, Ultrasound Workspace, TomTec Imaging Systems, Unterschleissheim, Germany). LV GLS was obtained in apical four- and two-chamber, and long-axis views in the apical, midline, and basal segments and RV values in a focused RV chamber view [26]. The normal reference ranges were defined by European Association of Cardiovascular Imaging (EACVI) guidelines: < -20% for LV GLS, < -23% for RV FWS [28], and < -21.5% for RV GLS [29]. Two independent investigators blinded to clinical data analyzed strain parameters, and interrater reliability was confirmed by reanalysing 20 randomly selected images.

### Clinical evaluation and cardiopulmonary exercise testing

In addition to TTE, all participants underwent comprehensive clinical evaluation, including medical history, physical examination, 12-lead electrocardiogram (ECG), blood sampling, bioimpedance analysis, hand grip force, and cardiopulmonary exercise testing (CPET). CPET was conducted on cycle ergometers (Excalibur Sport, LODE B.V., Groningen, The Netherlands) using a linear ramp protocol designed to achieve exhaustion within 8–12 min according to Clinical Recommendations for Cardiopulmonary Exercise Testing [30]. Breath-by-breath gas analysis (Quark CPET, Rome, Italy; MetaLyzer, CORTEX Biophysics, Leipzig, Germany; or Ergostik, Geratherm, Geratal, Germany) was performed to determine VO_2_max/kg, calculated as the mean oxygen uptake during the final 30 s of exercise.

Persistent symptoms, including wheezing, dyspnea, chest pain, and performance impairment such as CF or RPE were rated with “yes” if present and “no” if not present at the day of measurement using standardized questionnaires. Dyspnea was classified by the modified Medical Research Council (mMRC) dyspnea scale, a self-assessment test.

### Statistical analysis

Statistical analyses were performed using R version 4.3.0 (RRID: SCR_001905). Due to similarities in echocardiographic parameters within measures of one study center (intra-class correlation coefficient (ICC): [0.05; 0.91]), random effects linear regression models with intercepts varying across study centers and case-control status as a fixed variable were used (R-package lme4) to analyse differences between PCS and CON. Results were adjusted for potentially confounding variables (age, sex, BMI, diastolic blood pressure (BP), systolic BP, heart rate (HR), cardiac medication, and smoking [yes vs. never vs. lifetime smoking but currently not]). As effect size Cohens‘d was calculated based on t-values and degrees of freedom. To test symptom-specific differences in echocardiographic parameters, random effects linear regression models with intercepts varying across study centers and the presence of symptoms (yes vs. no; wheezing, dyspnea, chest pain, CF, RPE) as a fixed variable were performed. Results were adjusted for confounding. Associations between echocardiographic parameters and relative VO_2_max were conducted using random effects linear regression models with intercepts varying across study centers and LV GLS, RV GLS /RV FWS, TAPSE and diastolic function as fixed predictor variable. For all analyses, an alpha of 0.05 was considered as significant.

## Results

### Cohort characteristics

A total of 1558 individuals (982 participants PCS and 576 age- and gender-matched CON from Phase 1) presented themselves at four study centers between December 2021 and October 2022. For the analysis, 679 PCS (mean age 49 ± 12 years; 66% female) and 475 CON (mean age 48 ± 12 years; 66% female) were included (Fig. 1). Individuals with health status changes since Phase 1 (*n* = 404) were excluded. Sociodemographic and clinical characteristics are displayed in Table 1. The time since infection was comparable between groups (73±13 weeks in PCS and 72±14 weeks in CON), respectively. PCS participants had significantly higher rates of cardiovascular risk factors, including diabetes mellitus (4.4% vs. 1.1%, *p* = 0.002), obesity (30% vs. 13%, *p* < 0.001), smoking (8% vs. 4.2%, *p* < 0.001), and pre-existing cardiovascular conditions before (24% vs. 12%, *p* < 0.001) and after infection (9.2% vs. 1.9%, *p* < 0.001), as well as use of cardiac medication (31% vs. 15%, *p* < 0.001). During the acute infection, PCS were more frequently hospitalized (8.2% vs. 1.3%, *p* < 0.001) and medically treated (47% vs. 9.3%, *p* < 0.001).

**Table 1:** Cohort characteristics

	PCS, *N* = 679^1^	Controls, *N* = 474^1^	*p* value
Age, years *(M*,* SD)*	49 (12)	48 (12)	0.349
Sex male *(n*,* %)* female *(n*,* %)*	233/679 (34%) 446/679 (66%)	161/475 (34%) 314/475 (66%)	0.882
Nationality (German) *(n*,* %)*	663/679 (97.6%)	469 (98.7%)	0.130
BMI, kg/m^2^*(M*,* SD)*	28.0 (6.3)	25.0 (4.5)	< 0.001**
Body surface area, m^2^*(M*,* SD)*	1.93 (0.23)	1.86 (0.22)	< 0.001**
HR, bpm *(M*,* SD)*	68 (11)	67 (11)	0.078
Systolic BP, mmHg *(M*,* SD)*	129 (16)	128 (16)	0.261
Diastolic BP, mmHg *(M*,* SD)*	81 (10)	79 (9)	0.032
spO2, % *(M*,* SD)*	96.44 (7.18)	96.66 (9.14)	0.675
Cardiovascular Risk Factors *(n*,* %)*			
DM	30/679 (4.4%)	5/475 (1.1%)	0.002**
Obesity (≥ 30 kg/m2)	204/677 (30%)	62/474 (13%)	< 0.001**
Smoking			< 0.001**
No, no-regularly lifetime smoking	415/677 (61%)	357/475 (75%)	
Currently not, but lifetime-smoking	208/677 (31%)	98/475 (21%)	
Currently smoking	54/677 (8.0%)	20/475 (4.2%)	
Pre-existing cardiovascular disease *(n*,* %)*			< 0.001**
No	450/673 (67%)	409/475 (86%)	
Before SARS-CoV-2 infection	161/673 (24%)	57/475 (12%)	
After SARS-CoV-2 infection	62/673 (9.2%)	9/475 (1.9%)	
Myocardial Infarction	5 (0.7%)	0	
Congestive Heart Failure	9 (1.3%)	0	
PAD	2 (0.3%)	0	
Cerebrovascular Disease	15 (2.2%)	4 (0.8)	
Cardiac medication *(n*,* %)*	212/679 (31%)	73/475 (15%)	< 0.001**
Diuretics	44 (6.5%)	13 (2.7)	
Beta-blocker	72 (10.6%)	14 (2.9)	
ACE inhibitor/ARB	140 (20.6%)	54 (11.4)	
Calcium channel blocker	41 (6.0%)	19 (4.0)	
Statin	63 (9.3%)	29 (6.1)	
Vaccination Status *(n*,* %)*			0.009*
Not vaccinated	22/679 (3.2%)	29/475 (6.1%)	
1	73/679 (11%)	30/475 (6.3%)	
2	517/679 (76%)	371/475 (78%)	
3	67/679 (9.9%)	45/475 (9.5%)	
Recovered health condition *(M*,* SD)*	69.3% (16.0%)	100.0 (0.00)	< 0.001**
Recovered working capacity *(M*,* SD)*	68.5% (21.8%)	99.2 (5.9)	< 0.001**
Period between infection and study participation (weeks) *(M*,* SD)*	73 (13)	72 (14)	0.245
Symptoms *(n*,* %)*			
Dyspnoe (yes)	492/675 (73%)	46/475 (9.7%)	< 0.001**
mMRC scale			< 0.001**
0	140/488 (29%)	35/46 (76%)	
1	277/488 (57%)	11/46 (24%)	
2	50/488 (10%)	0/46 (0%)	
3	17/488 (3.5%)	0/46 (0%)	
4	4/488 (0.8%)	0/46 (0%)	
Whistling or Wheezing Breathing	159/679 (23%)	0/475 (0%)	< 0.001**
Chest Pain	171/679 (25%)	10/475 (2.1%)	< 0.001**
CF (yes)	478/678 (71%)	4/475 (0.8%)	< 0.001**
RPE (yes)	558/679 (82%)	10/475 (2.1%)	< 0.001**
Laboratory parameters *(M*,* SD)*			
Haemoglobin (g/dl)	14.12 (1.23)	14.03 (1.26)	0.234
SII	592 (417)	571 (533)	0.491
Creatine kinase (U/l)	139 (207)	157 (324)	0.277
Myoglobin (ng/ml)	42 (19)	46 (51)	0.131
pro-BNP (pg/ml)	85 (93)	83 (98)	0.718
Ferritin (µg/ml)	126 (139)	117 (149)	0.297

Abbreviations: PCS = post-COVID syndrome, BMI = body mass index, HR = heart rate, bpm = beats/min, BP = blood pressure, spO_2_ = oxygen saturation, DM = diabetes mellitus, EOD = end-organ damage, PAD = occlusive peripheral arterial disease, ACE inhibitor/ARB = angiontensin converting enzyme inhibitor/ angiotensin receptor blocker, ICU = intensive care unit, mMRC = Modified Medical Research Council, CF = chronic fatigue, RPE = rapid physical exhaustion, SII = systemic immune-inflammation index, pro-BNP = pro-brain natriuretic peptide

### Cardiac structure and function

**Fig. 2:**
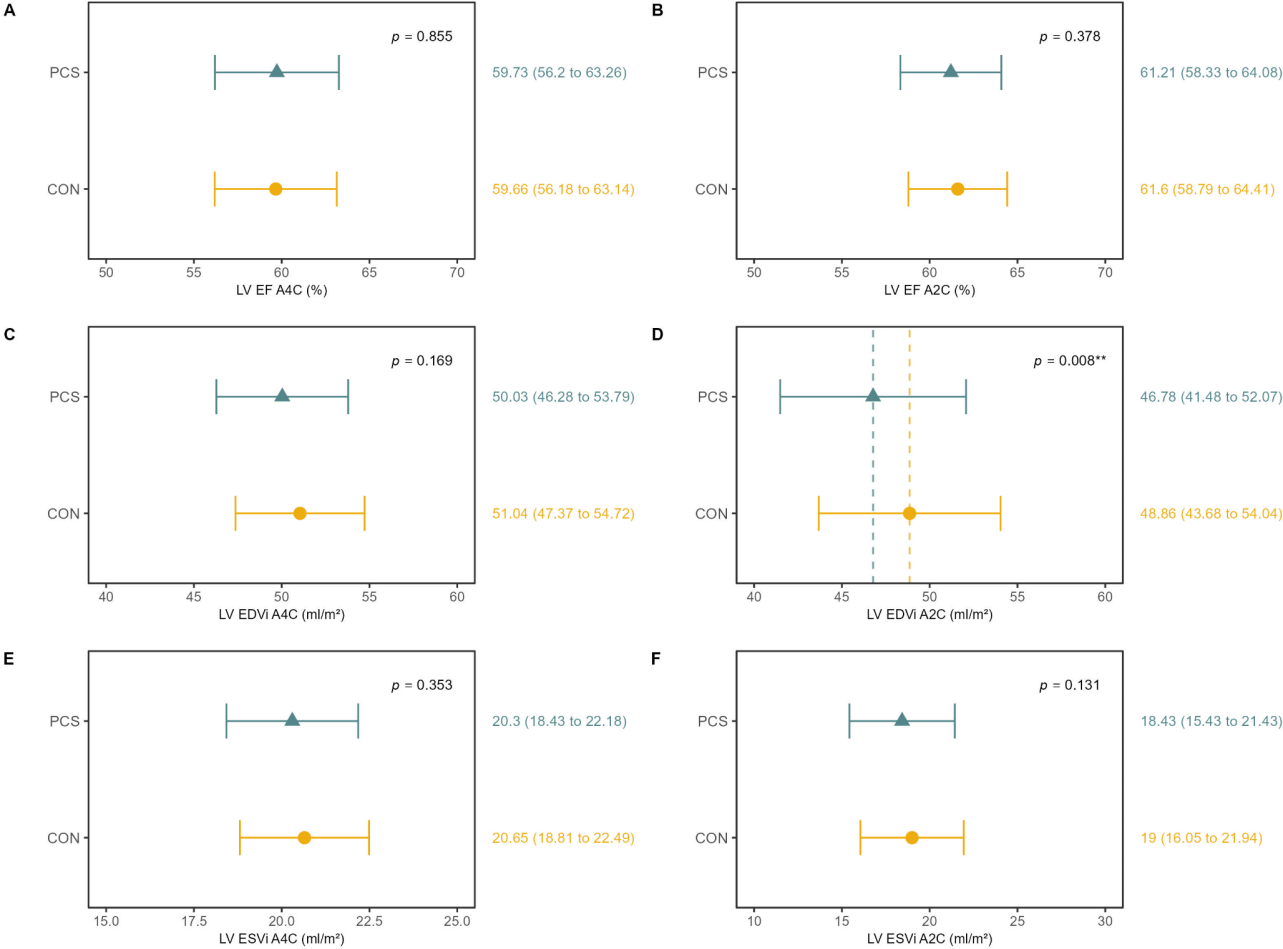
Echocardiographic Parameters of the Left Ventricle. The top panel shows adjusted means and 95% CI of the echocardiographic parameters recorded in the PCS and CON group 1.5 years after SARS-CoV-2 infection. Abbreviations: LV EF A4C = left ventricular ejection fraction in four-chamber view, A2C = two-chamber view, EDVi A4C = end-diastolic volume in four-chamber view indexed to body surface area, ESVi = end-systolic volume indexed to body surface area

**Fig. 3:**
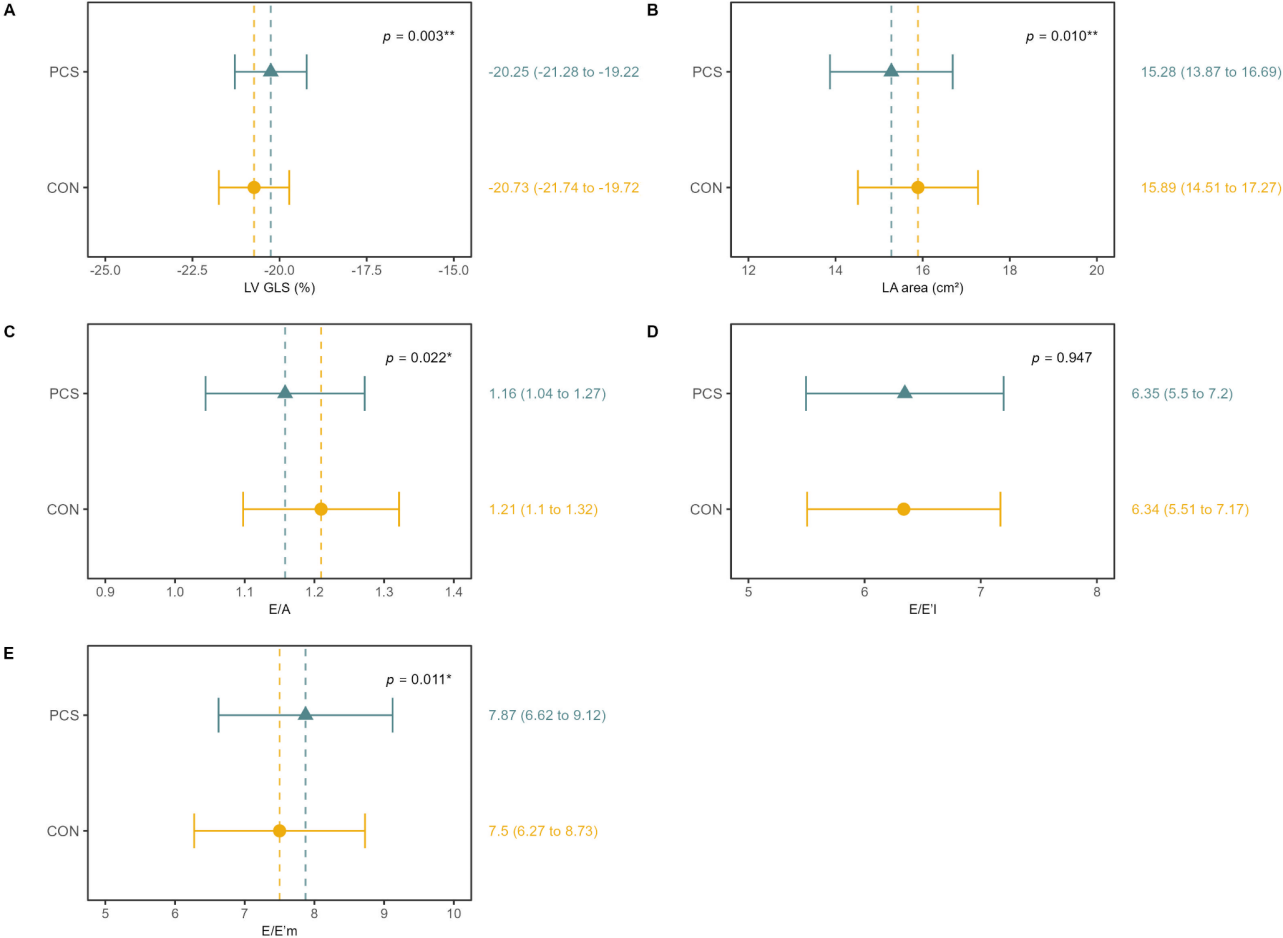
Echocardiographic Parameters of Diastolic Function and LV GLS. LV GLS = left ventricular global longitudinal strain, LA = left atrium, E/A = E/A ratio

**Fig. 4:**
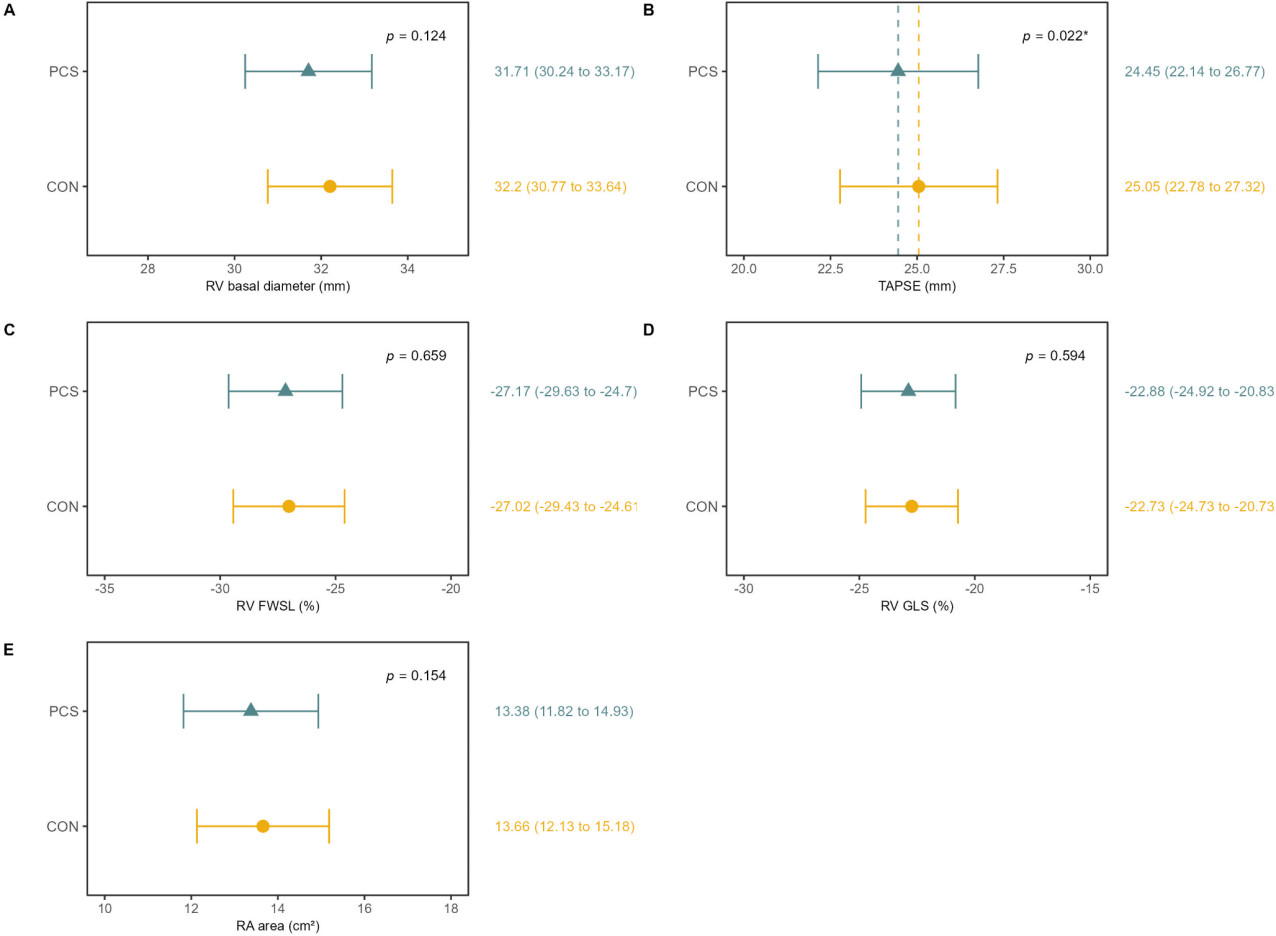
Echocardiographic Parameters of the Right Ventricle and RV GLS and RV FWS. RV = right ventricle, TAPSE = tricuspid annular plane systolic excursion, RV FWS = right ventricle free wall strain, RV GLS = right ventricle global longitudinal strain, RA = right atrium

Mean echocardiographic parameters for LV and RV structure and function were within normal limits for both groups [26]. However, PCS showed significantly lower LVEDVi A2C (46.78 ml/m^2^ [95%CI, 41.48–52.07] vs. 48.86 ml/m^2^ [95%CI, 43.68–54.04], *p* = 0.008, d = 0.11), E/A ratio (1.16 [95%CI, 1.04–1.27] vs. 1.21 [95%CI, 1.1–1.32], *p* = 0.022, d = 0.17) and E/E´m ratio (7.87 [95%CI, 6.62–9.12] vs. 7.5 [95%CI, 6.27–8.73], *p* = 0.011, d=-0.18) compared to CON (Figs. 2 and 3). Depending on the image quality, LV GLS could be evaluated in 739 participants (414 PCS and 324 CON), RV GLS in 791 participants (452 PCS and 339 CON), and RV FWS in 793 participants (454 PCS and 339 CON). LV GLS was reduced in PCS (-20.25% [95%CI, -21.28– -19.22] vs. -20.73% [95%CI, -21.74 – -19.72], *p* = 0.003, d=-0.22). Differences persisted after adjustment for confounders, including age, sex, BMI, HR, systolic or diastolic BP, cardiac medication, smoking status, and study center (Supplemental Table 2), and remained when individuals with pre-existing cardiovascular disease were excluded (Supplemental Table 3). A higher proportion of PCS had reduced LV GLS > -20% (PCS: 44% vs. CON: 36.3%, *p* = 0.031) (Supplemental Table 4). TAPSE was significantly lower in PCS than in CON (24.45 mm [95%CI, 22.14–26.77] vs. 25.05 mm [95%CI, 22.78–27.32], *p* = 0.022, d = 0.15) (Fig. 4). There were no significant group differences in RV GLS, RV FWS and RV dimensions, though a trend toward higher pathological RV FWS in PCS (PCS: 18.1% vs. CON: 12.7%, *p* = 0.050) (Supplemental Table 4). Interrater reliability concerning the LV GLS measure showed excellent agreement (Cronbachs Alpha: 0.94 [95%CI, 0.87–0.97]). Interrater reliability for RV GLS (Cronbachs Alpha: 0.73 [95%CI, 0.47–0.87] and RV FWS (Cronbachs Alpha: 0.60 [95%CI, 0.22–0.80] was moderate.

### Persisting symptoms after COVID-19

All persistent symptoms were significantly more prevalent in PCS than in CON (Table 1). The most common were RPE (82% vs. 2.1%, *p* < 0.001), dyspnea (73% vs. 9.7%, *p* < 0.001), and CF (71% vs. 0.8%, *p* < 0.001), followed by chest pain (25% vs. 2.1%, *p* < 0.001) and wheezing (23% vs. 0%, *p* < 0.001). PCS were also more likely to report mMRC grade 1 (57% vs. 24%, *p* < 0.001) or grades 2–4, which were absent in CON.

### Association of left and right ventricular function with relative VO2max and persisting symptoms

**Fig. 5:**
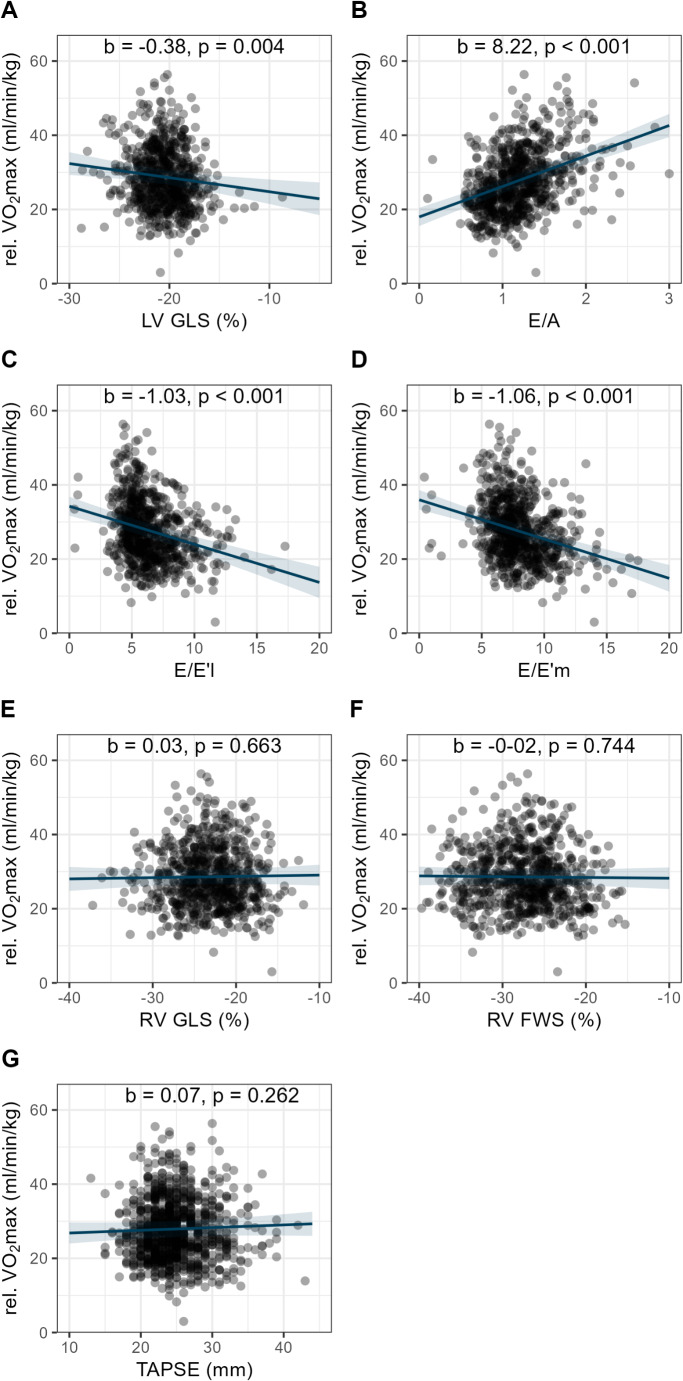
Association of Strain Parameters, TAPSE and Diastolic Function with VO_2_ max. The scatter plot illustrates the relationship between LV and RV GLS, RV FWS, TAPSE, and diastolic function with maximum oxygen uptake (= relative VO_2_max (ml/min/kg)). Abbreviations: LV GLS = left ventricular global longitudinal strain, E/A = E/A ratio, RV FWS = right ventricle free wall strain, RV GLS = right ventricle global longitudinal strain, TAPSE = tricuspid annular plane systolic excursion

In the combined cohort, LV GLS correlated negatively with relative VO_2_max (b=-0.38, t(712)=-2.93, *p* = 0.004), indicating worse systolic function with lower fitness. No significant associations were observed for RV GLS (b = 0.03, t(761) = 0.44, *p* = 0.663), RV FWS (b=-0.02, t(763)=-0.33, *p* = 0.744) or TAPSE (b = 0.07, t(971) = 1.12, *p* = 0.262) with relative VO_2_max. Diastolic function parameters showed significant associations with VO_2_max ((E/A (b = 8.22, t(758) = 11.46, *p* < 0.001); E/E´l (b=-1.03, t(756)=-7.25, *p* < 0.001), E/E´m (b=-1.06, t(758)=-7.84, *p* < 0.001)) (Supplemental Table 7, Fig. 5) and E/A ratio with the occurrence of chest pain (*p* = 0.040). LV GLS was positively associated with RPE (b = 0.51, t(398) = 1.97, *p* = 0.050), but not with wheezing, dyspnea, chest pain, or CF. RV strain parameters and TAPSE were not associated with any symptoms (Supplemental Table 6).

## Discussion

This multicenter cohort study evaluates long-term cardiac structure and function in individuals with post-COVID syndrome (PCS) compared to recovered controls (CON). While PCS was associated with subtle reductions in cardiac function and exercise capacity, the clinical significance of these findings warrants further investigation.

### Left ventricular function

The dimensions, systolic and diastolic function, and LV GLS in both groups were within normal reference ranges [26]. However, LV GLS was significantly reduced in PCS compared to CON, with a greater proportion of PCS patients exhibiting pathological values. This aligns with studies of hospitalized patients during the acute phase [11, 12] and recovery [14, 15, 16], reporting reduced LV GLS. For example, Lassen et al. observed reduced LV GLS during hospitalization and two months post-infection [23], and Baruch et al. reported abnormal LV GLS in 25% of patients three months after hospitalization [19]. Diastolic dysfunction was also more pronounced in PCS, consistent with studies correlating LV GLS and the E/A ratio in post-COVID patients [16]. In contrast, some follow-up studies, including those by Gao et al. and Baum et al., found no significant changes in LV GLS, except among hospitalized patients with pre-existing cardiovascular disease, highlighting a potential link between cardiovascular morbidities and impaired LV GLS [14, 20, 31]. Importantly, our cohort included PCS participants with a higher prevalence of pre-existing cardiovascular conditions and metabolic risk factors, such as overweight and diabetes. These comorbidities are known contributors to LV GLS impairment [32] and could partially account for the observed differences between groups. Adjusted linear regression analyses were employed to control for these confounders, and significantly reduced LV GLS persisted in PCS.

Proposed mechanisms for persistent cardiac dysfunction include complement and platelet activation [33], systemic inflammation [2, 4], endothelial dysfunction accompanied by coronary spasm [34], and mitochondrial impairment in cardiac and skeletal muscle [35]. Moreover, recent evidence highlights the role of endothelial dysfunction in cardiovascular risk among post-COVID patients up to 12 months post-infection [36]. These findings suggest that subclinical vascular alterations may contribute to cardiac dysfunction in PCS. In our cohort, subclinical LV changes correlated with reduced exercise capacity and VO_2_max, consistent with findings in ischemic heart disease [37] and heart failure [38]. Earlier studies of post-COVID patients provided limited evidence for this relationship [22, 39], however our results suggest that impaired LV function could contribute to exercise intolerance. This is evidenced by an inverse association between LV GLS and respiratory exchange ratio. Alternative explanations, such as deconditioning [40], or reduced pre-infection physical performance [41], cannot be excluded. While the observed reductions in LV GLS were statistically significant, their clinical relevance is uncertain as values remained within reference ranges. Further longitudinal studies are necessary to determine the long-term impact of these changes on clinical outcomes and to guide diagnostic and therapeutic strategies for PCS.

### RV remodeling and RV GLS/RV FWS

Two years post-infection, no significant differences in RV dimensions, RV GLS, or RV FWS were observed between PCS and CON groups. However, TAPSE was significantly reduced in PCS, albeit within normal limits. The low hospitalization (8.2%) and intensive care unit admission (1.6%) rates in our cohort likely explain the absence of significant RV remodeling, which has been primarily reported in severe COVID-19 cases with ARDS or mechanical ventilation [17, 18]. Similar findings have been reported in studies of non-hospitalized COVID-19 patients, where TAPSE reductions were noted without significant RV strain changes [42, 43]. TAPSE measures basal segment motion during systole but does not fully capture RV geometry [44], whereas RV GLS is a more sensitive marker for RV dysfunction [29]. The feasibility of RV GLS measurement was limited in our cohort, potentially underestimating early dysfunction. Overall, long-term impact of RV remodeling appears minimal in PCS and is likely restricted to a subset of patients with severe initial infections.

### Association of left and right ventricular function with persisting symptoms

The most commonly reported symptoms in PCS included RPE, dyspnea, and CF, consistent with other long-term studies [14, 24]. While no significant associations were found between LV GLS, RV GLS, RV FWS, or TAPSE and typical cardiopulmonary symptoms, subtle reductions in LV GLS were positively associated with RPE. These findings align with previous work [14, 20, 45], and emphasize the multifactorial nature of PCS and the need for comprehensive evaluation and symptom-based scoring to better characterize and manage these patients [46]. While myocardial scars identified by MRI studies appear to have limited clinical relevance for cardiopulmonary fitness post-infection [47], our results suggest that subtle reductions in LV GLS may partially underlie exercise impairments, as evidenced by the negative correlation between LV GLS and relative VO_2_max. Emerging evidence points to immunometabolic alterations and microvascular dysfunction as potential contributors to physical activity-induced post-exertional malaise in PCS and related conditions, such as myalgic encephalomyelitis/chronic fatigue syndrome [48]. However, other factors, including metabolic comorbidities such as overweight and diabetes, may also contribute to both cardiac dysfunction and symptom persistence.

### Strengths and limitations

This study`s strengths include its large, multicenter, population-based design focusing on working-age adults with confirmed SARS-CoV-2 infection. By minimizing the overrepresentation of severely ill, hospitalized, or elderly patients with comorbidities, the study improves the generalizability to broader, real-world populations. Comparing PCS patients to recovered controls, rather than uninfected individuals, reflects real-world conditions in largely exposed and now immunized population. The study`s rigorous methodology, including blinded cardiac imaging analyses and adjusted regression models, further enhances its reliability.

However, the lack of pre-COVID-19 echocardiographic data limits the ability to directly assess changes from pre-infection to the evaluation period. STE analysis, while robust, is limited by load dependency, re-test variability, and image quality. Selection bias is possible, as symptomatic individuals may have been more likely to participate. Moreover, the cohort`s demographic and geographic characteristics may limit generalizability to other populations. Adjusted linear regression was utilized to account for baseline differences in characteristics such as BMI, diabetes, and pre-existing cardiovascular disease, but residual bias cannot be entirely excluded. Future studies should incorporate pre-infection data, specifically investigate variant-specific impact on cardiac function, and apply advanced statistical methodologies to address confounding variables more effectively. Incorporating advanced imaging modalities, such as cardiac MRI, will enable better characterization of subclinical cardiac and vascular conditions and their implications for long-term outcomes in PCS patients.

## Conclusions

This study identifies subtle yet consistent reductions in LV function, particularly LV GLS, diastolic function, and exercise capacity in PCS compared to CON, with limited right ventricular involvement. However, the observed differences are minimal and within normal reference ranges, and their clinical relevance remains uncertain. Further research is needed to elucidate the relationship between persistent symptoms, cardiac involvement, and exercise intolerance to inform diagnostic and therapeutic strategies. These findings underscore the importance of comprehensive cardiac assessments and follow-up in symptomatic PCS patients while highlighting the need for further investigation to clarify the long-term implications of these observations.

## Electronic supplementary material

Below is the link to the electronic supplementary material.

Supplementary Material 1

## Data Availability

Some of the data generated during and/or analyzed during the current study are included in this published article and its supplementary information files. The data will be shared on reasonable request to the corresponding author.
