# Supplementary material for: Cardiac structure and function 1.5 years after COVID-19: results from the EPILOC study
Source: Infection. 2025 Feb 24;53(5):1685–97. doi: 10.1007/s15010-025-02481-4 (PMC12460391; doi:10.1007/s15010-025-02481-4)
Supplement: Supplementary file 1 — Supplementary Material 1 [file 15010_2025_2481_MOESM1_ESM.docx]

**Supplemental Material** **to**

**Cardiac Structure and Function 1.5 years After COVID-19:**

**Results from the EPILOC Study**

Jana Schellenberg MDa, Lynn Matits MSca, b, Daniel A. Bizjak PhDa, Peter Deibert MDc, Birgit Friedmann-Bette MDd, Siri Göpel MDe, Uta Merle MDf, Andreas Niess MDg, Norbert Frey MDh, Oliver Morathc, Gunnar Erz MDg, Raphael S. Peter PhDi, Alexandra Nieters PhDj, Dietrich Rothenbacher MDi, Winfried V. Kern MDk, Jürgen M. Steinacker MDa,l

Corresponding author: Dr. med. Jana Schellenberg, University Hospital Ulm, Sports and Rehabilitation Medicine, Email: jana.schellenberg@uniklinik-ulm.de

Supplemental Table 1 Ultrasound equipment page 2

Supplemental Table 2 Adjusted linear regression models (LMM) page 2

Supplemental Table 3 Adjusted linear regression models (LMM) for additional analyses without cardiovascular diseases page 9

Supplemental Table 4 Pathologic and non-pathologic strain values between post-COVID syndrome and recovered controls page 16

Supplemental Table 5 Differences in adjusted means between participants with post-COVID syndrome with certain symptoms page 16

Supplemental Table 6 Differences in echocardiographic parameters between participants with post-COVID syndrome with vs. without certain symptoms (adjusted) page 17

Supplemental Table 7 Association of relative VO_2_max with echocardiographic parameters page 25

**Supplemental Table 1 Ultrasound equipment**

| Study center | Number | Ultrasound machine | Transducer |
| --- | --- | --- | --- |
| Ulm | 318 | EPIQ Elite (Philips GmbH, Hamburg, Germany) | X5-1 |
| Freiburg | 299 | EPIQ 7c (Philips GmbH, Hamburg, Germany) | X5-1 |
| Heidelberg | 293 | Vivid E95 (GE, Vingmed Ultrasound, Horten, Norway) | 4Vc-D, M5Sc-D |
| Tübingen | 244 | Affiniti CVx (Philips GmbH, Hamburg, Germany) | XS5-1, L12-3 |

**Supplemental Table 2 Adjusted linear regression models (LMM)**

| Variable |  | estimate | | SE | | t | | df | | p | 95% CI | |
| --- | --- | --- | --- | --- | --- | --- | --- | --- | --- | --- | --- | --- |
| LV EF A4C | (Intercept) | 68.21 | | 2.27 | | 30.08 | | 984 | | <.001*** | [63.76, 72.66] | |
|  | Case | -0.06 | | 0.35 | | -0.18 | | 984 | | .855 | [-0.76, 0.63] | |
|  | Age | -0.01 | | 0.02 | | -0.77 | | 984 | | .443 | [-0.04, 0.02] | |
|  | Sex female | 1.85 | | 0.37 | | 5.02 | | 984 | | <.001*** | [1.13, 2.57] | |
|  | BMI | -0.13 | | 0.03 | | -3.65 | | 984 | | <.001*** | [-0.19, -0.06] | |
|  | Diastolic BP | -0.06 | | 0.03 | | -2.29 | | 984 | | .022* | [-0.11, -0.01] | |
|  | Systolic BP | 0.01 | | 0.02 | | 0.82 | | 984 | | .412 | [-0.02, 0.04] | |
|  | HR | -0.04 | | 0.02 | | -2.42 | | 984 | | .016* | [-0.07, -0.01] | |
|  | Cardiac Medication | 0.01 | | 0.42 | | 0.02 | | 984 | | .983 | [-0.82, 0.84] | |
|  | Lifetime Smoking | 0.52 | 0.39 | | 1.32 | | 984 | | .186 | | [-0.25, 1.28] |  |
|  | Currently Smoking | -0.29 | 0.69 | | -042 | | 984 | | .672 | | [-1.65, 1.07] |  |
|  | SD (Intercept) | 2.25 | 0.94 | |  | |  | |  | | [0.99, 5.11] |  |
|  | SD (Observations) | 5.21 | 0.12 | |  | |  | |  | | [4.98, 5.44] |  |
| AIC = 6177.58; AICc = 6177.95; BIC = 6241.35; R^2^ cond = 0.21; R^2^ marg = 0.08; ICC = 0.09; RMSE = 5.17 Sigma = 5.21 | | | | | | | | | | | |  |
| LV EF A2C | (Intercept) | 63.67 | 2.61 | | 24.37 | | 747 | | <.001*** | | [58.54, 68.80] |  |
|  | Case | 0.39 | 0.45 | | 0.88 | | 747 | | .378 | | [-0.48, 1.27] |  |
|  | Age | 0.02 | 0.02 | | 0.84 | | 747 | | .403 | | [-0.02, 0.05] |  |
|  | Sex female | 3.23 | 0.46 | | 6.99 | | 747 | | < .001*** | | [2.32, 4.14] |  |
|  | BMI | -0.08 | 0.04 | | -1.84 | | 747 | | .066 | | [-0.17, 0.01] |  |
|  | Diastolic BP | -0.03 | 0.03 | | -0.84 | | 747 | | .403 | | [-0.09, 0.03] |  |
|  | Systolic BP | 0.01 | 0.02 | | 0.68 | | 747 | | .495 | | [-0.03, 0.05] |  |
|  | HR | -0.04 | 0.02 | | -1.89 | | 747 | | .059 | | [-0.07, 0.00] |  |
|  | Cardiac Medication | -0.20 | 0.54 | | -0.37 | | 747 | | .713 | | [-1.25, 0.86] |  |
|  | Lifetime Smoking | 0.31 | 0.50 | | 0.63 | | 747 | | .531 | | [-0.66, 1.29] |  |
|  | Currently Smoking | 0.49 | 0.81 | | 0.60 | | 747 | | .546 | | [-1.10, 2.08] |  |
|  | SD (Intercept) | 1.82 | 0.80 | |  | |  | |  | | [0.77, 4.31] |  |
|  | SD (Observations) | 5.76 | 0.15 | |  | |  | |  | | [5.47, 6.05] |  |
| AIC = 4867.55; AICc = 4868.04; BIC = 4927.78; R^2^ cond = 0.16; R^2^ marg = 0.06; ICC = 0.16; RMSE = 5.70; Sigma = 5.76 | | | | | | | | | | | |  |
| LV EDVi A4C | (Intercept) | 63.46 | 4.18 | | 15.19 | | 750 | | <.001*** | | [55.26, 71.66] |  |
|  | Case | 1.01 | 0.73 | | 1.38 | | 750 | | .169 | | [-0.43, 2.45] |  |
|  | Age | -0.09 | 0.03 | | -3.00 | | 750 | | .003** | | [-0.15, -0.03] |  |
|  | Sex female | -8.24 | 0.76 | | -10.86 | | 750 | | <.001*** | | [-9.73, -6.75] |  |
|  | BMI | -0.02 | 0.07 | | -0.31 | | 750 | | .759 | | [-0.17, 0.12] |  |
|  | Diastolic BP | -0.03 | 0.05 | | -0.65 | | 750 | | .516 | | [-0.13, 0.07] |  |
|  | Systolic BP | 0.04 | 0.03 | | 1.15 | | 750 | | .249 | | [-0.03, 0.10] |  |
|  | HR | -0.09 | 0.03 | | -2.94 | | 750 | | .003** | | [-0.15, -0.03] |  |
|  | Cardiac Medication | 0.54 | 0.88 | | 0.62 | | 750 | | .537 | | [-1.19, 2.27] |  |
|  | Lifetime Smoking | -0.42 | 0.82 | | -0.51 | | 750 | | .611 | | [-2.02, 1.19] |  |
|  | Currently Smoking | -0.61 | 1.34 | | -0.46 | | 750 | | .649 | | [-3.25, 2.02] |  |
|  | SD (Intercept) | 2.36 | 1.08 | |  | |  | |  | | [0.96, 5.78] |  |
|  | SD (Observations) | 9.46 | 0.24 | |  | |  | |  | | [8.99, 9.95] |  |
| AIC = 5632.14; AICc = 5632.62; BIC = 5692.42; R^2^ cond = 0.21; R^2^ marg = 0.16; ICC = 0.06; RMSE = 9.37; Sigma = 9.46 | | | | | | | | | | | |  |
| LV EDVi A2C | (Intercept) | 72.14 | 4.58 | | 15.73 | | 739 | | <.001*** | | [63.13, 81.14] |  |
|  | Case | 2.08 | 0.78 | | 2.67 | | 739 | | .008** | | [0.55, 3.62] |  |
|  | Age | -0.17 | 0.03 | | -5.14 | | 739 | | <.001*** | | [-0.23, -0.10] |  |
|  | Sex female | -5.31 | 0.81 | | -6.58 | | 739 | | <.001*** | | [-6.90, -3.73] |  |
|  | BMI | -0.20 | 0.08 | | -2.55 | | 739 | | .011* | | [-0.35, -0.05] |  |
|  | Diastolic BP | -0.10 | 0.05 | | -1.78 | | 739 | | .076 | | [-0.20, 0.01] |  |
|  | Systolic BP | 0.05 | 0.03 | | 1.48 | | 739 | | .139 | | [-0.02, 0.12] |  |
|  | HR | -0.11 | 0.03 | | -3.38 | | 739 | | .001*** | | [-0.18, -0.05] |  |
|  | Cardiac Medication | -0.63 | 0.94 | | -0.66 | | 739 | | .507 | | [-2.48, 1.22] |  |
|  | Lifetime Smoking | -0.46 | 0.87 | | -0.52 | | 739 | | .600 | | [-2.16, 1.25] |  |
|  | Currently Smoking | 0.29 | 1.44 | | 0.20 | | 739 | | .838 | | [-2.53, 3.11] |  |
|  | SD (Intercept) | 3.36 | 1.46 | |  | |  | |  | | [1.43, 7.90] |  |
|  | SD (Observations) | 10.01 | 0.26 | |  | |  | |  | | [9.51, 10.53] |  |
| AIC = 5636.52; AICc = 5637.02; BIC = 5696.62; R^2^ cond = 0.22; R^2^ marg = 0.13; ICC = 0.10; RMSE = 9.91; Sigma = 10.01 | | | | | | | | | | | |  |
| LV ESVi A4C | (Intercept) | 21.44 | 2.12 | | 10.11 | | 750 | | <.001*** | | [17.28, 25.60] |  |
|  | Case | 0.35 | 0.37 | | 0.93 | | 750 | | .353 | | [-0.39, 1.08] |  |
|  | Age | -0.03 | 0.02 | | -2.09 | | 750 | | .037* | | [-0.06, -0.00] |  |
|  | Sex female | -4.36 | 0.39 | | -11.30 | | 750 | | <.001*** | | [-5.12, -3.60] |  |
|  | BMI | 0.04 | 0.04 | | 1.12 | | 750 | | .262 | | [-0.03, 0.11] |  |
|  | Diastolic BP | 0.02 | 0.03 | | 0.62 | | 750 | | .536 | | [-0.03, 0.07] |  |
|  | Systolic BP | 0.01 | 0.02 | | 0.42 | | 750 | | .675 | | [-0.03, 0.04] |  |
|  | HR | -0.01 | 0.02 | | -0.58 | | 750 | | .563 | | [-0.04, 0.02] |  |
|  | Cardiac Medication | 0.09 | 0.45 | | 0.20 | | 750 | | .841 | | [-0.79, 0.97] |  |
|  | Lifetime Smoking | -0.19 | 0.41 | | -0.45 | | 750 | | .653 | | [-1.00, 0.63] |  |
|  | Currently Smoking | -0.02 | 0.69 | | -0.03 | | 750 | | .977 | | [-1.37, 1.33] |  |
|  | SD (Intercept) | 1.18 | 0.54 | |  | |  | |  | | [0.48, 2.90] |  |
|  | SD (Observations) | 4.81 | 0.12 | |  | |  | |  | | [4.57, 5.06] |  |
| AIC = 4614.06; AICc = 4614.55; BIC = 4674.35; R^2^ cond = 0.21; R^2^ marg = 0.16; ICC = 0.06; RMSE = 4.76; Sigma = 4.81 | | | | | | | | | | | |  |
| LV ESVi A2C | (Intercept) | 28.06 | 2.26 | | 12.39 | | 739 | | <.001*** | | [23.62, 32.51] |  |
|  | Case | 0.57 | 0.38 | | 1.51 | | 739 | | .131 | | [-0.17, 1.31] |  |
|  | Age | -0.07 | 0.02 | | -4.20 | | 739 | | <.001*** | | [-0.10, -0.04] |  |
|  | Sex female | -3.66 | 0.39 | | -9.42 | | 739 | | <.001*** | | [-4.43, -2.90] |  |
|  | BMI | -0.06 | 0.04 | | -1.48 | | 739 | | .138 | | [-0.13, 0.02] |  |
|  | Diastolic BP | -0.02 | 0.03 | | -0.74 | | 739 | | .459 | | [-0.07, 0.03] |  |
|  | Systolic BP | 0.00 | 0.02 | | 0.23 | | 739 | | .822 | | [-0.03, 0.04] |  |
|  | HR | -0.03 | 0.02 | | -1.59 | | 739 | | .113 | | [-0.06, 0.01] |  |
|  | Cardiac Medication | -0.14 | 0.45 | | -0.31 | | 739 | | .753 | | [-1.03, 0.75] |  |
|  | Lifetime Smoking | -0.32 | 0.42 | | -0.76 | | 739 | | .450 | | [-1.14, 0.51] |  |
|  | Currently Smoking | -0.37 | 0.69 | | -0.53 | | 739 | | .596 | | [-1.72, 0.99] |  |
|  | SD (Intercept) | 1.90 | 0.82 | |  | |  | |  | | [0.82, 4.41] |  |
|  | SD (Observations) | 4.81 | 0.13 | |  | |  | |  | | [4.58, 5.07] |  |
| AIC = 4553.56; AICc = 4554.05; BIC = 4613.65; R^2^ cond = 0.25; R^2^ marg = 0.13; ICC = 0.14; RMSE = 4.77; Sigma = 4.81 | | | | | | | | | | | |  |
| LV GLS | (Intercept) | -26.19 | 1.02 | | -25.72 | | 720 | | <.001*** | | [-28.19, -24.19] |  |
|  | Case | -0.48 | 0.16 | | -2.94 | | 720 | | .003** | | [-0.80, -0.16] |  |
|  | Age | 0.02 | 0.01 | | 2.68 | | 720 | | .008** | | [0.01, 0.03] |  |
|  | Sex female | -0.97 | 0.17 | | -5.74 | | 720 | | <.001*** | | [-1.31, -0.64] |  |
|  | BMI | 0.00 | 0.02 | | 0.24 | | 720 | | .809 | | [-0.03, 0.04] |  |
|  | Diastolic BP | 0.03 | 0.01 | | 2.47 | | 720 | | .014* | | [0.01, 0.05] |  |
|  | Systolic BP | 0.01 | 0.01 | | 1.54 | | 720 | | .124 | | [-0.00, 0.03] |  |
|  | HR | 0.02 | 0.01 | | 3.19 | | 720 | | .002** | | [0.01, 0.04] |  |
|  | Cardiac Medication | 0.04 | 0.20 | | 0.19 | | 720 | | .850 | | [-0.35, 0.43] |  |
|  | Lifetime Smoking | -0.03 | 0.18 | | -0.14 | | 720 | | .886 | | [-0.39, 0.33] |  |
|  | Currently Smoking | -0.11 | 0.32 | | -0.34 | | 720 | | .733 | | [-0.73, 0.51] |  |
|  | SD (Intercept) | 0.66 | 0.29 | |  | |  | |  | | [0.28, 1.55] |  |
|  | SD (Observations) | 2.08 | 0.05 | |  | |  | |  | | [1.97, 2.19] |  |
| AIC = 3222.81; AICc = 3223.32; BIC = 3282.58; R^2^ cond = 0.21; R^2^ marg = 0.13; ICC = 0.09; RMSE = 2.06; Sigma = 2.08 | | | | | | | | | | | |  |
| LA diam | (Intercept) | 33.01 | 1.82 | | 18.17 | | 1,002 | | <.001*** | | [29.44, 36.57] |  |
|  | Case | 0.32 | 0.28 | | 1.12 | | 1,002 | | .261 | | [-0.24, 0.88] |  |
|  | Age | 0.03 | 0.01 | | 2.27 | | 1,002 | | .024* | | [0.00, 0.05] |  |
|  | Sex female | -2.62 | 0.30 | | -8.88 | | 1,002 | | <.001*** | | [-3.20, -2.04] |  |
|  | BMI | 0.26 | 0.03 | | 9.70 | | 1,002 | | <.001*** | | [0.21, 0.32] |  |
|  | Diastolic BP | -0.04 | 0.02 | | -1.90 | | 1,002 | | .057 | | [-0.08, 0.00] |  |
|  | Systolic BP | 0.02 | 0.01 | | 1.79 | | 1,002 | | .073 | | [-0.00, 0.05] |  |
|  | HR | -0.08 | 0.01 | | -6.22 | | 1,002 | | <.001*** | | [-0.10, -0.05] |  |
|  | Cardiac Medication | 1.38 | 0.34 | | 4.10 | | 1,002 | | <.001*** | | [0.72, 2.04] |  |
|  | Lifetime Smoking | 0.00 | 0.31 | | 0.00 | | 1,002 | | .997 | | [-0.61, 0.61] |  |
|  | Currently Smoking | -0.39 | 0.56 | | -0.70 | | 1,002 | | .484 | | [-1.48, 0.70] |  |
|  | SD (Intercept) | 1.85 |  | |  | |  | |  | |  |  |
|  | SD (Observations) | 4.21 |  | |  | |  | |  | |  |  |
| AIC = 5860.52; AICc = 5860.89; BIC = 5924.52; R^2^ cond = 0.36; R^2^ marg = 0.24; ICC = 0.16; RMSE = 4.18; Sigma = 4.21 | | | | | | | | | | | |  |
| LA area | (Intercept) | 13.45 | 1.36 | | 9.89 | | 771 | | <.001*** | | [10.78, 16.12] |  |
|  | Case | 0.61 | 0.24 | | 2.59 | | 771 | | .010** | | [0.15, 1.07] |  |
|  | Age | 0.01 | 0.01 | | 0.52 | | 771 | | .602 | | [-0.01, 0.02] |  |
|  | Sex female | -1.54 | 0.24 | | -6.31 | | 771 | | <.001*** | | [-2.02, -1.06] |  |
|  | BMI | 0.21 | 0.02 | | 9.11 | | 771 | | <.001*** | | [0.17, 0.26] |  |
|  | Diastolic BP | -0.03 | 0.02 | | -1.88 | | 771 | | .060 | | [-0.06, 0.00] |  |
|  | Systolic BP | 0.02 | 0.01 | | 1.88 | | 771 | | .061 | | [-0.00, 0.04] |  |
|  | Heart Rate | -0.05 | 0.01 | | -4.86 | | 771 | | <.001*** | | [-0.07, -0.03] |  |
|  | Cardiac Medication | 0.04 | 0.28 | | 0.15 | | 771 | | .884 | | [-0.51, 0.59] |  |
|  | Lifetime Smoking | -0.13 | 0.26 | | -0.48 | | 771 | | .630 | | [-0.64, 0.39] |  |
|  | Currently Smoking | 0.01 | 0.43 | | 0.01 | | 771 | | .988 | | [-0.84, 0.85] |  |
|  | SD (Intercept) | 0.89 | 0.40 | |  | |  | |  | | [0.37, 2.13] |  |
|  | SD (Observations) | 3.08 | 0.08 | |  | |  | |  | | [2.93, 3.24] |  |
| AIC = 4051.92; AICc = 4052.39; BIC = 4112.56; R^2^ cond = 0.25; R^2^ marg = 0.19; ICC = 0.08; RMSE = 3.05; Sigma = 3.08 | | | | | | | | | | | |  |
| E/A | (Intercept) | 2.89 | 0.13 | | 22.75 | | 774 | | <.001*** | | [2.64, 3.14] |  |
|  | Case | 0.05 | 0.02 | | 2.30 | | 774 | | .022* | | [0.01, 0.10] |  |
|  | Age | -0.02 | 0.00 | | -16.19 | | 774 | | <.001*** | | [-0.02, -0.01] |  |
|  | Sex female | 0.00 | 0.02 | | 0.13 | | 774 | | .898 | | [-0.04, 0.05] |  |
|  | BMI | -0.01 | 0.00 | | -4.10 | | 774 | | <.001*** | | [-0.01, -0.00] |  |
|  | Diastolic BP | -0.00 | 0.00 | | -2.36 | | 774 | | .019* | | [-0.01, -0.00] |  |
|  | Systolic BP | -0.00 | 0.00 | | -0.20 | | 774 | | .842 | | [-0.00, 0.00] |  |
|  | HR | -0.01 | 0.00 | | -6.19 | | 774 | | <.001*** | | [-0.01, -0.00] |  |
|  | Cardiac Medication | -0.01 | 0.03 | | -0.21 | | 774 | | .834 | | [-0.06, 0.05] |  |
|  | Lifetime Smoking | -0.02 | 0.02 | | -0.75 | | 774 | | .454 | | [-0.07, 0.03] |  |
|  | Currently Smoking | -0.03 | 0.04 | | -0.79 | | 774 | | .429 | | [-0.11, 0.05] |  |
|  | SD (Intercept) | 0.07 | 0.03 | |  | |  | |  | | [0.03, 0.18] |  |
|  | SD (Observations) | 0.29 | 0.01 | |  | |  | |  | | [0.28, 0.31] |  |
| AIC = 418.20; AICc = 418.67; BIC = 478.88; R^2^ cond = 0.41; R^2^ marg = 0.37; ICC = 0.06; RMSE = 0.29; Sigma = 0.29 | | | | | | | | | | | |  |
| E/E´l | (Intercept) | 1.20 | 0.79 | | 1.53 | | 771 | | .127 | | [-0.34, 2.74] |  |
|  | Case | -0.01 | 0.14 | | -0.07 | | 771 | | .947 | | [-0.28, 0.26] |  |
|  | Age | 0.05 | 0.01 | | 9.46 | | 771 | | <.001*** | | [0.04, 0.07] |  |
|  | Sex female | 0.26 | 0.14 | | 1.88 | | 771 | | .060 | | [-0.01, 0.54] |  |
|  | BMI | 0.04 | 0.01 | | 2.96 | | 771 | | .003** | | [0.01, 0.07] |  |
|  | Diastolic BP | -0.00 | 0.01 | | -0.02 | | 771 | | .987 | | [-0.02, 0.02] |  |
|  | Systolic BP | 0.01 | 0.01 | | 1.29 | | 771 | | .198 | | [-0.00, 0.02] |  |
|  | HR | 0.00 | 0.01 | | 0.66 | | 771 | | .512 | | [-0.01, 0.02] |  |
|  | Cardiac Medication | 0.30 | 0.16 | | 1.86 | | 771 | | .063 | | [-0.02, 0.62] |  |
|  | Lifetime Smoking | 0.01 | 0.15 | | 0.03 | | 771 | | .973 | | [-0.29, 0.30] |  |
|  | Currently Smoking | -0.13 | 0.25 | | -0.51 | | 771 | | .610 | | [-0.62, 0.37] |  |
|  | SD (Intercept) | 0.54 | 0.24 | |  | |  | |  | | [0.23, 1.28] |  |
|  | SD (Observations) | 1.77 | 0.05 | |  | |  | |  | | [1.68, 1.86] |  |
| AIC = 3196.44; AICc = 3196.92; BIC = 3257.08; R^2^ cond = 0.24; R^2^ marg = 0.17; ICC = 0.09; RMSE = 1.75; Sigma = 1.77 | | | | | | | | | | | |  |
| E/E`m | (Intercept) | 3.13 | 0.89 | | 3.53 | | 773 | | <.001*** | | [1.39, 4.88] |  |
|  | Case | -0.37 | 0.15 | | -2.55 | | 773 | | .011* | | [-0.66, -0.09] |  |
|  | Age | 0.05 | 0.01 | | 7.47 | | 773 | | <.001*** | | [0.03, 0.06] |  |
|  | Sex female | 0.46 | 0.15 | | 3.05 | | 773 | | .002** | | [0.16, 0.76] |  |
|  | BMI | 0.04 | 0.01 | | 2.88 | | 773 | | .004** | | [0.01, 0.07] |  |
|  | Diastolic BP | -0.01 | 0.01 | | -0.91 | | 773 | | .363 | | [-0.03, 0.01] |  |
|  | Systolic BP | 0.01 | 0.01 | | 2.30 | | 773 | | .022* | | [0.00, 0.03] |  |
|  | HR | 0.00 | 0.01 | | 0.18 | | 773 | | .855 | | [-0.01, 0.01] |  |
|  | Cardiac Medication | 0.04 | 0.17 | | 0.23 | | 773 | | .822 | | [-0.30, 0.38] |  |
|  | Lifetime Smoking | -0.24 | 0.16 | | -1.47 | | 773 | | .141 | | [-0.56, 0.08] |  |
|  | Currently Smoking | 0.02 | 0.27 | | 0.08 | | 773 | | .934 | | [-0.51, 0.55] |  |
|  | SD (Intercept) | 0.79 | 0.34 | |  | |  | |  | | [0.34, 1.82] |  |
|  | SD (Observations) | 1.91 | 0.05 | |  | |  | |  | | [1.82, 2.01] |  |
| AIC = 3323.53; AICc = 3324.00; BIC = 3384.20; R^2^ cond = 0.25; R^2^ marg = 0.12; ICC = 0.15; RMSE = 1.89; Sigma = 1.91 | | | | | | | | | | | |  |
| RV basal | (Intercept) | 34.49 | 1.81 | | 19.00 | | 761 | | <.001*** | | [30.93, 38.05] |  |
|  | Case | 0.50 | 0.32 | | 1.54 | | 761 | | .124 | | [-0.14, 1.13] |  |
|  | Age | 0.03 | 0.01 | | 1.89 | | 761 | | .060 | | [-0.00, 0.05] |  |
|  | Sex female | -3.19 | 0.33 | | -9.57 | | 761 | | <.001*** | | [-3.85, -2.54] |  |
|  | BMI | 0.07 | 0.03 | | 2.25 | | 761 | | .025* | | [0.01, 0.13] |  |
|  | Diastolic BP | 0.00 | 0.02 | | 0.04 | | 761 | | .970 | | [-0.04, 0.04] |  |
|  | Systolic BP | -0.01 | 0.01 | | -0.49 | | 761 | | .625 | | [-0.03, 0.02] |  |
|  | HR | -0.05 | 0.01 | | -3.27 | | 761 | | .001** | | [-0.07, -0.02] |  |
|  | Cardiac Medication | -0.53 | 0.39 | | -1.39 | | 761 | | .166 | | [-1.29, 0.22] |  |
|  | Lifetime Smoking | -0.86 | 0.36 | | -2.39 | | 761 | | .017* | | [-1.56, -0.15] |  |
|  | Currently Smoking | 0.34 | 0.59 | | 0.57 | | 761 | | .569 | | [-0.82, 1.50] |  |
|  | SD (Intercept) | 0.91 | 0.43 | |  | |  | |  | | [0.36, 2.29] |  |
|  | SD (Observations) | 4.19 | 0.11 | |  | |  | |  | | [3.99, 4.41] |  |
| AIC = 4471.57; AICc = 4472.05; BIC = 4532.04; R^2^ cond = 0.18; R^2^ marg = 0.14; ICC = 0.05; RMSE = 4.16; Sigma = 4.19 | | | | | | | | | | | |  |
| RV mid | (Intercept) | 26.44 | 2.32 | | 11.38 | | 624 | | <.001*** | | [21.88, 31.01] |  |
|  | Case | 0.13 | 0.35 | | 0.37 | | 624 | | .714 | | [-0.56, 0.81] |  |
|  | Age | -0.03 | 0.01 | | -1.95 | | 624 | | .051 | | [-0.06, 0.00] |  |
|  | Sex female | -3.00 | 0.36 | | -8.33 | | 624 | | <.001*** | | [-3.71, -2.30] |  |
|  | BMI | 0.04 | 0.03 | | 1.09 | | 624 | | .274 | | [-0.03, 0.11] |  |
|  | Diastolic BP | -0.01 | 0.02 | | -0.36 | | 624 | | .715 | | [-0.06, 0.04] |  |
|  | Systolic BP | 0.01 | 0.02 | | 0.54 | | 624 | | .589 | | [-0.02, 0.04] |  |
|  | HR | -0.04 | 0.01 | | -2.63 | | 624 | | .009** | | [-0.07, -0.01] |  |
|  | Cardiac Medication | -0.46 | 0.43 | | -1.07 | | 624 | | .286 | | [-1.30, 0.38] |  |
|  | Lifetime Smoking | -0.00 | 0.39 | | -0.00 | | 624 | | 1.00 | | [-0.77, 0.77] |  |
|  | Currently Smoking | -0.15 | 0.61 | | -0.24 | | 624 | | .810 | | [-1.34, 1.05] |  |
|  | SD (Intercept) | 2.67 | 1.12 | |  | |  | |  | | [1.17, 6.08] |  |
|  | SD (Observations) | 4.10 | 0.12 | |  | |  | |  | | [3.88, 4.33] |  |
| AIC = 3664.40; AICc = 3664.98; BIC = 3722.33; R^2^ cond = 0.36; R^2^ marg = 0.09; ICC = 0.30; RMSE = 4.05; Sigma = 4.10 | | | | | | | | | | | |  |
| RV length | (Intercept) | 41.60 | 15.93 | | 2.61 | | 624 | | .009** | | [10.32, 72.88] |  |
|  | Case | 0.52 | 0.81 | | 0.63 | | 624 | | .526 | | [-1.08, 2.11] |  |
|  | Age | -0.06 | 0.03 | | -1.81 | | 624 | | .070 | | [-0.13, 0.01] |  |
|  | Sex female | -4.77 | 0.84 | | -5.69 | | 624 | | <.001*** | | [-6.41, -3.12] |  |
|  | BMI | 0.28 | 0.08 | | 3.52 | | 624 | | <.001*** | | [0.13, 0.44] |  |
|  | Diastolic BP | 0.01 | 0.06 | | 0.25 | | 624 | | .806 | | [-0.10, 0.12] |  |
|  | Systolic BP | 0.01 | 0.04 | | 0.36 | | 624 | | .717 | | [-0.06, 0.08] |  |
|  | HR | -0.01 | 0.03 | | -0.34 | | 624 | | .736 | | [-0.08, 0.05] |  |
|  | Cardiac Medication | -0.61 | 1.00 | | -0.61 | | 624 | | .541 | | [-2.56, 1.35] |  |
|  | Lifetime Smoking | -0.64 | 0.91 | | -0.70 | | 624 | | .485 | | [-2.43, 1.15] |  |
|  | Currently Smoking | 0.03 | 1.42 | | 0.02 | | 624 | | .981 | | [-2.75, 2.82] |  |
|  | SD (Intercept) | 30.60 | 12.51 | |  | |  | |  | | [13.74, 68.19] |  |
|  | SD (Observations) | 9.55 | 0.27 | |  | |  | |  | | [9.03, 10.09] |  |
| AIC = 4732.45; AICc = 4733.04; BIC = 4790.39; R^2^ cond = 0.91; R^2^ marg = 0.01; ICC = 0.91; RMSE = 9.44; Sigma = 9.55 | | | | | | | | | | | |  |
| TAPSE | (Intercept) | 25.96 | 1.61 | | 16.12 | | 995 | | <.001*** | | [22.80, 29.12] |  |
|  | Case | 0.59 | 0.26 | | 2.29 | | 995 | | .022* | | [0.09, 1.10] |  |
|  | Age | -0.01 | 0.01 | | -0.54 | | 995 | | .588 | | [-0.03, 0.02] |  |
|  | Sex female | 0.05 | 0.27 | | 0.19 | | 995 | | .850 | | [-0.48, 0.58] |  |
|  | BMI | 0.10 | 0.02 | | 4.20 | | 995 | | <.001*** | | [0.06, 0.15] |  |
|  | Diastolic BP | -0.06 | 0.02 | | -3.39 | | 995 | | .001*** | | [-0.10, -0.03] |  |
|  | Systolic BP | 0.03 | 0.01 | | 2.53 | | 995 | | .012* | | [0.01, 0.05] |  |
|  | HR | -0.04 | 0.01 | | -3.39 | | 995 | | .001*** | | [-0.06, -0.02] |  |
|  | Cardiac Medication | -0.06 | 0.31 | | -0.21 | | 995 | | .835 | | [-0.67, 0.54] |  |
|  | Lifetime Smoking | -0.20 | 0.29 | | -0.70 | | 995 | | .482 | | [-0.76, 0.36] |  |
|  | Currently Smoking | -0.18 | 0.51 | | -0.35 | | 995 | | .724 | | [-1.18, 0.82] |  |
|  | SD (Intercept) | 1.48 |  | |  | |  | |  | |  |  |
|  | SD (Observations) | 3.83 |  | |  | |  | |  | |  |  |
| AIC = 5633.96; AICc = 5634.33; BIC = 5697.87; R^2^ cond = 0.16; R^2^ marg = 0.04; ICC = 0.13; RMSE = 3.81; Sigma = 3.83 | | | | | | | | | | | |  |
| RV FWS | (Intercept) | -30.29 | 2.05 | | -14.78 | | 774 | | <.001*** | | [-34.32, -26.27] |  |
|  | Case | 0.15 | 0.34 | | 0.44 | | 774 | | .659 | | [-0.51, 0.81] |  |
|  | Age | -0.01 | 0.01 | | -0.85 | | 774 | | .395 | | [-0.04, 0.02] |  |
|  | Sex female | -1.50 | 0.35 | | -4.27 | | 774 | | <.001*** | | [-2.19, -0.81] |  |
|  | BMI | 0.07 | 0.03 | | 1.94 | | 774 | | .053 | | [-0.00, 0.13] |  |
|  | Diastolic BP | 0.02 | 0.02 | | 0.82 | | 774 | | .410 | | [-0.03, 0.07] |  |
|  | Systolic BP | 0.00 | 0.02 | | 0.27 | | 774 | | .788 | | [-0.03, 0.03] |  |
|  | HR | 0.01 | 0.02 | | 0.55 | | 774 | | .585 | | [-0.02, 0.04] |  |
|  | Cardiac Medication | 0.33 | 0.41 | | 0.80 | | 774 | | .427 | | [-0.48, 1.14] |  |
|  | Lifetime Smoking | 0.02 | 0.38 | | 0.05 | | 774 | | .962 | | [-0.73, 0.76] |  |
|  | Currently Smoking | -0.44 | 0.65 | | -0.68 | | 774 | | .497 | | [-1.72, 0.83] |  |
|  | SD (Intercept) | 1.58 | 0.67 | |  | |  | |  | | [0.68, 3.64] |  |
|  | SD (Observations) | 4.43 | 0.11 | |  | |  | |  | | [4.22, 4.66] |  |
| AIC = 4635.05; AICc = 4635.53; BIC = 4695.74; R^2^ cond = 0.15; R^2^ marg = 0.04; ICC = 0.11; RMSE = 4.39; Sigma = 4.43 | | | | | | | | | | | |  |
| RV GLS | (Intercept) | -24.91 | 1.67 | | -14.88 | | 772 | | <.001*** | | [-28.20, -21.62] |  |
|  | Case | 0.15 | 0.27 | | 0.53 | | 772 | | .594 | | [-0.39, 0.68] |  |
|  | Age | 0.01 | 0.01 | | 1.12 | | 772 | | .264 | | [-0.01, 0.04] |  |
|  | Sex female | -1.59 | 0.29 | | -5.54 | | 772 | | <.001*** | | [-2.15, -1.02] |  |
|  | BMI | -0.00 | 0.03 | | -0.04 | | 772 | | .972 | | [-0.06, 0.05] |  |
|  | Diastolic BP | 0.04 | 0.02 | | 1.87 | | 772 | | .062 | | [-0.00, 0.08] |  |
|  | Systolic BP | -0.01 | 0.01 | | -0.91 | | 772 | | .362 | | [-0.04, 0.01] |  |
|  | HR | 0.01 | 0.01 | | 0.60 | | 772 | | .552 | | [-0.02, 0.03] |  |
|  | Cardiac Medication | 0.52 | 0.34 | | 1.53 | | 772 | | .127 | | [-0.15, 1.18] |  |
|  | Lifetime Smoking | 0.08 | 0.31 | | 0.24 | | 772 | | .807 | | [-0.53, 0.68] |  |
|  | Currently Smoking | -0.26 | 0.53 | | -0.49 | | 772 | | .625 | | [-1.30, 0.78] |  |
|  | SD (Intercept) | 1.31 | 0.56 | |  | |  | |  | | [0.57, 3.02] |  |
|  | SD (Observations) | 3.61 | 0.09 | |  | |  | |  | | [3.43, 3.79] |  |
| AIC = 4304.68; AICc = 4305.15; BIC = 4365.33; R^2^ cond = 0.16; R^2^ marg = 0.05; ICC = 0.12; RMSE = 3.58; Sigma = 3.61 | | | | | | | | | | | |  |
| RA area | (Intercept) | 14.87 | 1.18 | | 12.64 | | 764 | | <.001*** | | [12.56, 17.18] |  |
|  | Case | 0.28 | 0.20 | | 1.43 | | 764 | | .154 | | [-0.10, 0.66] |  |
|  | Age | 0.03 | 0.01 | | 3.15 | | 764 | | .002** | | [0.01, 0.04] |  |
|  | Sex female | -2.26 | 0.20 | | -11.16 | | 764 | | <.001*** | | [-2.65, -1.86] |  |
|  | BMI | 0.10 | 0.02 | | 5.11 | | 764 | | <.001*** | | [0.06, 0.14] |  |
|  | Diastolic BP | -0.01 | 0.01 | | -0.55 | | 764 | | .585 | | [-0.03, 0.02] |  |
|  | Systolic BP | 0.00 | 0.01 | | 0.22 | | 764 | | .828 | | [-0.01, 0.02] |  |
|  | HR | -0.05 | 0.01 | | -6.11 | | 764 | | <.001*** | | [-0.07, -0.03] |  |
|  | Cardiac Medication | -0.54 | 0.23 | | -2.33 | | 764 | | .020* | | [-1.00, -0.09] |  |
|  | Lifetime Smoking | -0.30 | 0.22 | | -1.37 | | 764 | | .171 | | [-0.73, 0.13] |  |
|  | Currently Smoking | -0.22 | 0.36 | | -0.63 | | 764 | | .532 | | [-0.92, 0.48] |  |
|  | SD (Intercept) | 0.99 | 0.42 | |  | |  | |  | | [0.43, 2.29] |  |
|  | SD (Observations) | 2.54 | 0.07 | |  | |  | |  | | [2.42, 2.67] |  |
| AIC = 3724.96; AICc = 3725.44; BIC = 3785.48; R^2^ cond = 0.32; R^2^ marg = 0.21; ICC = 0.13; RMSE = 2.52; Sigma = 2.54 | | | | | | | | | | | |  |

Note. Linear regression models (LMM) using case as predictor; controlled for age (per year), sex, BMI (per kg/m²), diastolic and systolic blood pressure (per mmHg) and heart rate (per bpm), smoking, center. Uncertainty intervals (equal-tailed) and p-values (two-tailed).

Abbreviations: LV EF A4C = left ventricular ejection fraction in four-chamber view, BMI = body mass index, HR = heart rate, A2C = two-chamber view, EDVi A4C = end-diastolic volume in four-chamber view indexed to body surface area, ESVi = end-systolic volume indexed to body surface area, LV GLS = left ventricular global longitudinal strain, LA diam = left atrium diameter, RV = right ventricle, mid = midventricular, E/A = E/A ratio, RV = right ventricle, TAPSE = tricuspid annular plane systolic excursion, RV FWS = right ventricle free wall strain, RV GLS = right ventricle global longitudinal strain, RA = right atrium

**Supplemental Table 3 Adjusted linear regression models (LMM) for additional analyses without cardiovascular diseases**

| Variable |  | estimate | SE | t | df | p | 95% CI |
| --- | --- | --- | --- | --- | --- | --- | --- |
| LV EF A4C | (Intercept) | 68.96 | 2.43 | 28.39 | 790 | < .001*** | [64.20, 73.73] |
|  | Case | -0.00 | 0.38 | -0.01 | 790 | .994 | [-0.75, 0.74] |
|  | Age | -0.00 | 0.02 | -0.09 | 790 | .925 | [-0.03, 0.03] |
|  | Sex female | 1.53 | 0.42 | 3.67 | 790 | < .001*** | [0.71, 2.35] |
|  | BMI | -0.13 | 0.04 | -3.17 | 790 | .002** | [-0.21, -0.05] |
|  | Diastolic BP | -0.09 | 0.03 | -3.11 | 790 | .002** | [-0.15, -0.03] |
|  | Systolic BP | 0.02 | 0.02 | 1.16 | 790 | .246 | [-0.01, 0.06] |
|  | HR | -0.03 | 0.02 | -1.81 | 790 | .071 | [-0.07, 0.00] |
|  | Cardiac Medication | -0.20 | 0.60 | -0.33 | 790 | .739 | [-1.39, 0.98] |
|  | Lifetime Smoking | 0.60 | 0.44 | 1.37 | 790 | .172 | [-0.26, 1.47] |
|  | Currently Smoking | -0.39 | 0.77 | -0.51 | 790 | .613 | [-1.91, 1.13] |
|  | SD (Intercept) | 2.28 | 0.96 |  |  |  | [1.00, 5.18] |
|  | SD (Observations) | 5.11 | 0.13 |  |  |  | [4.86, 5.36] |
| AIC = 4952.21; AICc = 4952.68; BIC = 5013.16; R^2^ cond = 0.21; R^2^ marg = 0.06; ICC = 0.17; RMSE = 5.06; Sigma = 5.11 | | | | | | | |
| LV EF A2C | (Intercept) | 62.32 | 2.83 | 22.01 | 608 | < .001*** | [56.76, 67.88] |
|  | Case | 0.73 | 0.48 | 1.53 | 608 | .126 | [-0.21, 1.67] |
|  | Age | 0.04 | 0.02 | 1.92 | 608 | .055 | [-0.00, 0.08] |
|  | Sex female | 2.90 | 0.52 | 5.63 | 608 | < .001*** | [1.89, 3.92] |
|  | BMI | -0.08 | 0.05 | -1.63 | 608 | .103 | [-0.18, 0.02] |
|  | Diastolic BP | -0.03 | 0.04 | -0.71 | 608 | .477 | [-0.09, 0.04] |
|  | Systolic BP | 0.00 | 0.02 | 0.16 | 608 | .872 | [-0.04, 0.05] |
|  | HR | -0.01 | 0.02 | -0.66 | 608 | .508 | [-0.05, 0.03] |
|  | Cardiac Medication | 0.65 | 0.75 | 0.88 | 608 | .380 | [-0.81, 2.12] |
|  | Lifetime Smoking | 0.28 | 0.56 | 0.50 | 608 | .619 | [-0.82, 1.37] |
|  | Currently Smoking | 0.87 | 0.90 | 0.97 | 608 | .332 | [-0.89, 2.64] |
|  | SD (Intercept) | 1.85 | 0.82 |  |  |  | [0.78, 4.41] |
|  | SD (Observations) | 5.64 | 0.16 |  |  |  | [5.33, 5.97] |
| AIC = 3958.35; AICc = 3958.95; BIC = 4015.96; R^2^ cond = 0.16; R^2^ marg = 0.07; ICC = 0.10; RMSE = 5.58; Sigma = 5.64 | | | | | | | |
| LV EDVi A4C | (Intercept) | 59.83 | 4.48 | 13.35 | 609 | < .001*** | [51.03, 68.63] |
|  | Case | 0.76 | 0.78 | 0.98 | 609 | .329 | [-0.77, 2.28] |
|  | Age | -0.12 | 0.03 | -3.72 | 609 | < .001*** | [-0.18, -0.06] |
|  | Sex female | -7.32 | 0.84 | -8.71 | 609 | < .001*** | [-8.97, -5.67] |
|  | BMI | 0.02 | 0.08 | 0.22 | 609 | .825 | [-0.14, 0.18] |
|  | Diastolic BP | -0.01 | 0.06 | -0.24 | 609 | .807 | [-0.13, 0.10] |
|  | Systolic BP | 0.06 | 0.04 | 1.57 | 609 | .118 | [-0.01, 0.13] |
|  | HR | -0.11 | 0.03 | -3.11 | 609 | .002** | [-0.17, -0.04] |
|  | Cardiac Medication | -0.95 | 1.21 | -0.78 | 609 | .434 | [-3.33, 1.43] |
|  | Lifetime Smoking | 0.27 | 0.91 | 0.29 | 609 | .770 | [-1.52, 2.05] |
|  | Currently Smoking | -0.84 | 1.46 | -0.57 | 609 | .568 | [-3.71, 2.03] |
|  | SD (Intercept) | 2.32 | 1.08 |  |  |  | [0.93, 5.78] |
|  | SD (Observations) | 9.17 | 0.26 |  |  |  | [8.67, 9.70] |
| AIC = 4557.88; AICc = 4558.47; BIC = 4615.50; R^2^ cond = 0.20; R^2^ marg = 0.15; ICC = 0.06; RMSE = 9.07; Sigma = 9.17 | | | | | | | |
| LV EDVi A2C | (Intercept) | 71.78 | 5.02 | 14.31 | 603 | < .001*** | [61.93, 81.63] |
|  | Case | 1.97 | 0.84 | 2.34 | 603 | .020* | [0.32, 3.63] |
|  | Age | -0.20 | 0.04 | -5.64 | 603 | < .001*** | [-0.27, -0.13] |
|  | Sex female | -4.60 | 0.91 | -5.03 | 603 | < .001*** | [-6.39, -2.80] |
|  | BMI | -0.14 | 0.09 | -1.56 | 603 | .119 | [-0.31, 0.04] |
|  | Diastolic BP | -0.14 | 0.06 | -2.31 | 603 | .021* | [-0.27, -0.02] |
|  | Systolic BP | 0.08 | 0.04 | 2.08 | 603 | .038* | [0.00, 0.16] |
|  | HR | -0.13 | 0.04 | -3.42 | 603 | .001*** | [-0.20, -0.05] |
|  | Cardiac Medication | -1.46 | 1.32 | -1.11 | 603 | .270 | [-4.06, 1.13] |
|  | Lifetime Smoking | 0.31 | 0.99 | 0.32 | 603 | .752 | [-1.63, 2.25] |
|  | Currently Smoking | 0.18 | 1.58 | 0.11 | 603 | .910 | [-2.93, 3.29] |
|  | SD (Intercept) | 3.45 | 1.52 |  |  |  | [1.46, 8.17] |
|  | SD (Observations) | 9.93 | 0.29 |  |  |  | [9.38, 10.50] |
| AIC = 4611.31; AICc = 4611.91; BIC = 4668.81; R^2^ cond = 0.22; R^2^ marg = 0.13; ICC = 0.11; RMSE = 9.82; Sigma = 9.93 | | | | | | | |
| LV ESVi A4C | (Intercept) | 20.26 | 2.25 | 8.98 | 610 | < .001*** | [15.83, 24.68] |
|  | Case | 0.19 | 0.39 | 0.48 | 610 | .630 | [-0.57, 0.95] |
|  | Age | -0.05 | 0.02 | -3.36 | 610 | .001*** | [-0.09, -0.02] |
|  | Sex female | -3.68 | 0.42 | -8.73 | 610 | < .001*** | [-4.50, -2.85] |
|  | BMI | 0.06 | 0.04 | 1.43 | 610 | .154 | [-0.02, 0.14] |
|  | Diastolic BP | 0.02 | 0.03 | 0.78 | 610 | .433 | [-0.03, 0.08] |
|  | Systolic BP | 0.02 | 0.02 | 1.14 | 610 | .256 | [-0.02, 0.06] |
|  | HR | -0.02 | 0.02 | -1.46 | 610 | .144 | [-0.06, 0.01] |
|  | Cardiac Medication | -0.11 | 0.61 | -0.18 | 610 | .860 | [-1.30, 1.09] |
|  | Lifetime Smoking | 0.14 | 0.45 | 0.31 | 610 | .753 | [-0.75, 1.03] |
|  | Currently Smoking | -0.01 | 0.73 | -0.01 | 610 | .991 | [-1.45, 1.43] |
|  | SD (Intercept) | 1.24 | 0.57 |  |  |  | [0.51, 3.06] |
|  | SD (Observations) | 4.59 | 0.13 |  |  |  | [4.34, 4.86] |
| AIC = 3719.07; AICc = 3719.67; BIC = 3719.67; R^2^ cond = 0.21; R^2^ marg = 0.12; ICC = 0.15; RMSE = 4.54; Sigma = 4.59 | | | | | | | |
| LV ESVi A2C | (Intercept) | 27.96 | 2.46 | 11.38 | 603 | < .001*** | [23.14, 32.78] |
|  | Case | 0.46 | 0.40 | 1.15 | 603 | .250 | [-0.33, 1.25] |
|  | Age | -0.09 | 0.02 | -5.17 | 603 | < .001*** | [-0.12, -0.05] |
|  | Sex female | -3.15 | 0.44 | -7.23 | 603 | < .001*** | [-4.01, -2.30] |
|  | BMI | -0.03 | 0.04 | -0.78 | 603 | .437 | [-0.12, 0.05] |
|  | Diastolic BP | -0.03 | 0.03 | -0.89 | 603 | .371 | [-0.08, 0.03] |
|  | Systolic BP | 0.02 | 0.02 | 0.88 | 603 | .381 | [-0.02, 0.05] |
|  | HR | -0.04 | 0.02 | -2.33 | 603 | .020* | [-0.08, -0.01] |
|  | Cardiac Medication | -0.44 | 0.63 | -0.69 | 603 | .489 | [-1.68, 0.80] |
|  | Lifetime Smoking | -0.08 | 0.47 | -0.17 | 603 | .865 | [-1.01, 0.85] |
|  | Currently Smoking | -0.53 | 0.76 | -0.70 | 603 | .485 | [-2.01, 0.96] |
|  | SD (Intercept) | 1.97 | 0.85 |  |  |  | [0.85, 4.58] |
|  | SD (Observations) | 4.74 | 0.14 |  |  |  | [4.48, 5.01] |
| AIC = 3717.45; AICc = 3718.05; BIC = 3774.95; R^2^ cond = 0.25; R^2^ marg = 0.13; ICC = 0.11; RMSE = 4.68; Sigma = 4.74 | | | | | | | |
| LV GLS | (Intercept) | -25.98 | 1.09 | -23.79 | 582 | < .001*** | [-28.12, -23.83] |
|  | Case | -0.58 | 0.18 | -3.25 | 582 | .001** | [-0.93, -0.23] |
|  | Age | 0.02 | 0.01 | 2.02 | 582 | .044* | [0.00, 0.03] |
|  | Sex female | -0.93 | 0.19 | -4.81 | 582 | < .001*** | [-1.31, -0.55] |
|  | BMI | 0.01 | 0.02 | 0.37 | 582 | .710 | [-0.03, 0.05] |
|  | Diastolic BP | 0.04 | 0.01 | 2.68 | 582 | .008** | [0.01, 0.06] |
|  | Systolic BP | 0.01 | 0.01 | 1.00 | 582 | .319 | [-0.01, 0.03] |
|  | HR | 0.02 | 0.01 | 2.40 | 582 | .017* | [0.00, 0.04] |
|  | Cardiac Medication | -0.14 | 0.29 | -0.47 | 582 | .637 | [-0.71, 0.44] |
|  | Lifetime Smoking | -0.04 | 0.21 | -0.21 | 582 | .833 | [-0.45, 0.36] |
|  | Currently Smoking | -0.03 | 0.35 | -0.08 | 582 | .933 | [-0.72, 0.66] |
|  | SD (Intercept) | 0.62 | 0.27 |  |  |  | [0.26, 1.48] |
|  | SD (Observations) | 2.05 | 0.06 |  |  |  | [1.94, 2.17] |
| AIC = 2611.01; AICc = 2611.64; BIC = 2668.06; R^2^ cond = 0.20; R^2^ marg = 0.13; ICC = 0.08; RMSE = 2.03; Sigma = 2.05 | | | | | | | |
| LA diameter | (Intercept) | 32.87 | 2.02 | 16.26 | 799 | < .001*** | [28.90, 36.83] |
|  | Case | 0.27 | 0.30 | 0.89 | 799 | .373 | [-0.33, 0.87] |
|  | Age | 0.03 | 0.01 | 2.28 | 799 | .023* | [0.00, 0.05] |
|  | Sex female | -2.48 | 0.33 | -7.51 | 799 | < .001*** | [-3.13, -1.83] |
|  | BMI | 0.26 | 0.03 | 8.10 | 799 | < .001*** | [0.19, 0.32] |
|  | Diastolic BP | -0.04 | 0.02 | -1.67 | 799 | .095 | [-0.08, 0.01] |
|  | Systolic BP | 0.03 | 0.01 | 1.83 | 799 | .068 | [-0.00, 0.05] |
|  | HR | -0.08 | 0.01 | -5.95 | 799 | < .001*** | [-0.11, -0.05] |
|  | Cardiac Medication | 0.87 | 0.47 | 1.84 | 799 | .066 | [-0.06, 1.80] |
|  | Lifetime Smoking | -0.48 | 0.35 | -1.36 | 799 | .173 | [-1.17, 0.21] |
|  | Currently Smoking | -0.10 | 0.61 | -0.16 | 799 | .873 | [-1.30, 1.11] |
|  | SD (Intercept) | 2.20 | 0.91 |  |  |  | [0.97, 4.97] |
|  | SD (Observations) | 4.08 | 0.10 |  |  |  | [3.89, 4.29] |
| AIC = 4650.04; AICc = 4650.49; BIC = 4711.13; R^2^ cond = 0.37; R^2^ marg = 0.18; ICC = 0.22; RMSE = 4.05; Sigma = 4.08 | | | | | | | |
| LA area | (Intercept) | 12.74 | 1.46 | 8.71 | 622 | < .001*** | [9.87, 15.62] |
|  | Case | 0.76 | 0.25 | 3.06 | 622 | .002** | [0.27, 1.25] |
|  | Age | -0.00 | 0.01 | -0.47 | 622 | .637 | [-0.03, 0.02] |
|  | Sex female | -1.30 | 0.27 | -4.81 | 622 | < .001*** | [-1.83, -0.77] |
|  | BMI | 0.24 | 0.03 | 9.01 | 622 | < .001*** | [0.19, 0.29] |
|  | Diastolic BP | -0.02 | 0.02 | -1.23 | 622 | .220 | [-0.06, 0.01] |
|  | Systolic BP | 0.02 | 0.01 | 1.76 | 622 | .079 | [-0.00, 0.04] |
|  | HR | -0.06 | 0.01 | -5.12 | 622 | < .001*** | [-0.08, -0.03] |
|  | Cardiac Medication | -0.46 | 0.39 | -1.18 | 622 | .237 | [-1.21, 0.30] |
|  | Lifetime Smoking | -0.31 | 0.29 | -1.08 | 622 | .280 | [-0.89, 0.26] |
|  | Currently Smoking | 0.16 | 0.47 | 0.34 | 622 | .730 | [-0.76, 1.08] |
|  | SD (Intercept) | 0.91 | 0.41 |  |  |  | [0.38, 2.18] |
|  | SD (Observations) | 2.98 | 0.08 |  |  |  | [2.82, 3.15] |
| AIC = 3248.95; AICc = 3249.53; BIC = 3306.84; R^2^ cond = 0.26; R^2^ marg = 0.17; ICC = 0.08; RMSE = 2.94; Sigma = 2.98 | | | | | | | |
| E/A | (Intercept) | 2.94 | 0.14 | 21.73 | 625 | < .001*** | [2.68, 3.21] |
|  | Case | 0.05 | 0.02 | 2.06 | 625 | .040* | [0.00, 0.09] |
|  | Age | -0.02 | 0.00 | -16.94 | 625 | < .001*** | [-0.02, -0.01] |
|  | Sex female | 0.04 | 0.03 | 1.77 | 625 | .077 | [-0.00, 0.09] |
|  | BMI | -0.01 | 0.00 | -3.84 | 625 | < .001*** | [-0.01, -0.00] |
|  | Diastolic BP | -0.00 | 0.00 | -1.90 | 625 | .058 | [-0.01, 0.00] |
|  | Systolic BP | -0.00 | 0.00 | -0.20 | 625 | .843 | [-0.00, 0.00] |
|  | HR | -0.01 | 0.00 | -6.68 | 625 | < .001*** | [-0.01, -0.00] |
|  | Cardiac Medication | 0.01 | 0.04 | 0.28 | 625 | .782 | [-0.06, 0.08] |
|  | Lifetime Smoking | -0.01 | 0.03 | -0.43 | 625 | .666 | [-0.06, 0.04] |
|  | Currently Smoking | -0.00 | 0.04 | -0.07 | 625 | .943 | [-0.09, 0.08] |
|  | SD (Intercept) | 0.08 | 0.03 |  |  |  | [0.03, 0.18] |
|  | SD (Observations) | 0.28 | 0.01 |  |  |  | [0.26, 0.29] |
| AIC = 290.89; AICc = 291.47; BIC = 348.85; R^2^ cond = 0.46; R^2^ marg = 0.42; ICC = 0.07; RMSE = 0.28; Sigma = 0.28 | | | | | | | |
| E/E´l | (Intercept) | 1.39 | 0.80 | 1.72 | 621 | .086 | [-0.20, 2.97] |
|  | Case | -0.14 | 0.14 | -1.01 | 621 | .314 | [-0.41, 0.13] |
|  | Age | 0.05 | 0.01 | 8.69 | 621 | < .001*** | [0.04, 0.06] |
|  | Sex female | 0.40 | 0.15 | 2.75 | 621 | .006** | [0.12, 0.69] |
|  | BMI | 0.03 | 0.01 | 2.08 | 621 | .038* | [0.00, 0.06] |
|  | Diastolic BP | -0.00 | 0.01 | -0.21 | 621 | .834 | [-0.02, 0.02] |
|  | Systolic BP | 0.01 | 0.01 | 1.60 | 621 | .110 | [-0.00, 0.02] |
|  | HR | 0.00 | 0.01 | 0.72 | 621 | .473 | [-0.01, 0.02] |
|  | Cardiac Medication | 0.29 | 0.21 | 1.38 | 621 | .168 | [-0.12, 0.70] |
|  | Lifetime Smoking | 0.05 | 0.16 | 0.34 | 621 | .734 | [-0.26, 0.37] |
|  | Currently Smoking | -0.13 | 0.26 | -0.49 | 621 | .622 | [-0.65, 0.39] |
|  | SD (Intercept) | 0.50 | 0.22 |  |  |  | [0.21, 1.20] |
|  | SD (Observations) | 1.63 | 0.05 |  |  |  | [1.54, 1.72] |
| AIC = 2492.95; AICc = 2493.54; BIC = 2550.82; R^2^ cond = 0.24; R^2^ marg = 0.21; ICC = 0.09; RMSE = 1.61; Sigma = 1.63 | | | | | | | |
| E/E´m | (Intercept) | 2.84 | 0.94 | 3.04 | 622 | .002** | [1.01, 4.68] |
|  | Case | -0.42 | 0.15 | -2.79 | 622 | .006** | [-0.72, -0.12] |
|  | Age | 0.04 | 0.01 | 6.40 | 622 | < .001*** | [0.03, 0.05] |
|  | Sex female | 0.63 | 0.16 | 3.85 | 622 | < .001*** | [0.31, 0.95] |
|  | BMI | 0.03 | 0.02 | 2.09 | 622 | .037* | [0.00, 0.06] |
|  | Diastolic BP | -0.01 | 0.01 | -1.13 | 622 | .258 | [-0.03, 0.01] |
|  | Systolic BP | 0.02 | 0.01 | 3.05 | 622 | .002** | [0.01, 0.04] |
|  | HR | 0.00 | 0.01 | 0.27 | 622 | .790 | [-0.01, 0.02] |
|  | Cardiac Medication | -0.06 | 0.23 | -0.26 | 622 | .799 | [-0.51, 0.40] |
|  | Lifetime Smoking | -0.21 | 0.18 | -1.19 | 622 | .234 | [-0.56, 0.14] |
|  | Currently Smoking | -0.12 | 0.29 | -0.41 | 622 | .685 | [-0.69, 0.45] |
|  | SD (Intercept) | 0.78 | 0.33 |  |  |  | [0.34, 1.81] |
|  | SD (Observations) | 1.81 | 0.05 |  |  |  | [1.71, 1.91] |
| AIC = 2628.13; AICc = 2628.72; BIC = 2686.03; R^2^ cond = 0.26; R^2^ marg = 0.12; ICC = 0.16; RMSE = 1.79; Sigma = 1.81 | | | | | | | |
| RV basal | (Intercept) | 34.91 | 1.97 | 17.75 | 617 | < .001*** | [31.05, 38.77] |
|  | Case | 0.35 | 0.35 | 1.00 | 617 | .316 | [-0.33, 1.04] |
|  | Age | 0.03 | 0.01 | 1.72 | 617 | .085 | [-0.00, 0.05] |
|  | Sex female | -3.48 | 0.38 | -9.23 | 617 | < .001*** | [-4.22, -2.74] |
|  | BMI | 0.10 | 0.04 | 2.83 | 617 | .005** | [0.03, 0.18] |
|  | Diastolic BP | 0.01 | 0.03 | 0.44 | 617 | .657 | [-0.04, 0.06] |
|  | Systolic BP | -0.02 | 0.02 | -1.01 | 617 | .312 | [-0.05, 0.02] |
|  | HR | -0.05 | 0.02 | -3.42 | 617 | .001*** | [-0.08, -0.02] |
|  | Cardiac Medication | -1.03 | 0.54 | -1.90 | 617 | .057 | [-2.10, 0.03] |
|  | Lifetime Smoking | -0.97 | 0.41 | -2.39 | 617 | .017* | [-1.77, -0.17] |
|  | Currently Smoking | 1.15 | 0.66 | 1.73 | 617 | .083 | [-0.15, 2.45] |
|  | SD (Intercept) | 0.69 | 0.37 |  |  |  | [0.24, 1.97] |
|  | SD (Observations) | 4.16 | 0.12 |  |  |  | [3.93, 4.40] |
| AIC = 3634.67; AICc = 3635.26; BIC = 3692.47; R^2^ cond = 0.19; R^2^ marg = 0.17; ICC = 0.03; RMSE = 4.11; Sigma = 4.16 | | | | | | | |
| RV mid | (Intercept) | 27.17 | 2.46 | 11.06 | 516 | < .001*** | [22.34, 31.99] |
|  | Case | -0.05 | 0.37 | -0.12 | 516 | .902 | [-0.78, 0.68] |
|  | Age | -0.03 | 0.02 | -1.77 | 516 | .078 | [-0.06, 0.00] |
|  | Sex female | -3.23 | 0.40 | -8.06 | 516 | < .001*** | [-4.01, -2.44] |
|  | BMI | 0.05 | 0.04 | 1.22 | 516 | .223 | [-0.03, 0.12] |
|  | Diastolic BP | -0.01 | 0.03 | -0.53 | 516 | .600 | [-0.07, 0.04] |
|  | Systolic BP | 0.01 | 0.02 | 0.54 | 516 | .586 | [-0.02, 0.04] |
|  | HR | -0.04 | 0.02 | -2.72 | 516 | .007** | [-0.07, -0.01] |
|  | Cardiac Medication | -0.65 | 0.58 | -1.11 | 516 | .268 | [-1.79, 0.50] |
|  | Lifetime Smoking | -0.09 | 0.44 | -0.20 | 516 | .839 | [-0.95, 0.77] |
|  | Currently Smoking | 0.39 | 0.68 | 0.58 | 516 | .563 | [-0.94, 1.73] |
|  | SD (Intercept) | 2.58 | 1.09 |  |  |  | [1.13, 5.92] |
|  | SD (Observations) | 4.06 | 0.13 |  |  |  | [3.82, 4.31] |
| AIC = 3039.02; AICc = 3039.73; BIC = 3094.54; R^2^ cond = 0.37; R^2^ marg = 0.11; ICC = 0.29; RMSE = 4.00; Sigma = 4.06 | | | | | | | |
| RV length | (Intercept) | 39.15 | 15.86 | 2.47 | 515 | .014* | [7.99, 70.30] |
|  | Case | 0.61 | 0.89 | 0.69 | 515 | .491 | [-1.13, 2.36] |
|  | Age | -0.06 | 0.04 | -1.69 | 515 | .092 | [-0.13, 0.01] |
|  | Sex female | -4.84 | 0.96 | -5.05 | 515 | < .001*** | [-6.72, -2.96] |
|  | BMI | 0.31 | 0.09 | 3.34 | 515 | .001*** | [0.13, 0.49] |
|  | Diastolic BP | 0.02 | 0.06 | 0.33 | 515 | .745 | [-0.10, 0.15] |
|  | Systolic BP | 0.02 | 0.04 | 0.47 | 515 | .636 | [-0.06, 0.10] |
|  | HR | 0.00 | 0.04 | 0.05 | 515 | .960 | [-0.07, 0.08] |
|  | Cardiac Medication | -0.16 | 1.40 | -0.12 | 515 | .906 | [-2.91, 2.58] |
|  | Lifetime Smoking | -0.61 | 1.05 | -0.59 | 515 | .559 | [-2.67, 1.44] |
|  | Currently Smoking | 1.17 | 1.63 | 0.72 | 515 | .472 | [-2.03, 4.37] |
|  | SD (Intercept) | 30.10 | 12.31 |  |  |  | [13.51, 67.09] |
|  | SD (Observations) | 9.71 | 0.30 |  |  |  | [9.13, 10.32] |
| AIC = 3944.77; AICc = 3945.48; BIC = 4000.27; R^2^ cond = 0.91; R^2^ marg = 0.01; ICC = 0.91; RMSE = 9.58; Sigma = 9.71 | | | | | | | |
| TAPSE | (Intercept) | 26.95 | 1.67 | 16.17 | 796 | < .001*** | [23.68, 30.22] |
|  | Case | 0.46 | 0.28 | 1.66 | 796 | .098 | [-0.08, 1.00] |
|  | Age | -0.00 | 0.01 | -0.37 | 796 | .710 | [-0.03, 0.02] |
|  | Sex female | 0.14 | 0.30 | 0.47 | 796 | .636 | [-0.45, 0.73] |
|  | BMI | 0.05 | 0.03 | 1.84 | 796 | .066 | [-0.00, 0.11] |
|  | Diastolic BP | -0.04 | 0.02 | -1.94 | 796 | .053 | [-0.08, 0.00] |
|  | Systolic BP | 0.02 | 0.01 | 1.63 | 796 | .103 | [-0.00, 0.05] |
|  | HR | -0.05 | 0.01 | -3.58 | 796 | < .001*** | [-0.07, -0.02] |
|  | Cardiac Medication | -0.26 | 0.43 | -0.60 | 796 | .552 | [-1.10, 0.59] |
|  | Lifetime Smoking | -0.66 | 0.32 | -2.07 | 796 | .039* | [-1.29, -0.03] |
|  | Currently Smoking | -0.77 | 0.56 | -1.37 | 796 | .170 | [-1.88, 0.33] |
|  | SD (Intercept) | 1.25 | 0.54 |  |  |  | [0.54, 2.90] |
|  | SD (Observations) | 3.71 | 0.09 |  |  |  | [3.53, 3.89] |
| AIC = 4476.65; AICc = 4477.11; BIC = 4537.70; R^2^ cond = 0.13; R^2^ marg = 0.03; ICC = 0.10; RMSE = 3.68; Sigma = 3.71 | | | | | | | |
| RV FWS | (Intercept) | -29.75 | 2.17 | -13.71 | 632 | < .001*** | [-34.02, -25.49] |
|  | Case | 0.15 | 0.36 | 0.41 | 632 | .680 | [-0.56, 0.86] |
|  | Age | -0.01 | 0.02 | -0.86 | 632 | .389 | [-0.04, 0.02] |
|  | Sex female | -1.29 | 0.40 | -3.22 | 632 | .001** | [-2.07, -0.50] |
|  | BMI | 0.11 | 0.04 | 2.71 | 632 | .007** | [0.03, 0.18] |
|  | Diastolic BP | -0.00 | 0.03 | -0.09 | 632 | .929 | [-0.06, 0.05] |
|  | Systolic BP | 0.01 | 0.02 | 0.58 | 632 | .563 | [-0.02, 0.05] |
|  | HR | -0.00 | 0.02 | -0.10 | 632 | .918 | [-0.03, 0.03] |
|  | Cardiac Medication | -0.08 | 0.60 | -0.13 | 632 | .896 | [-1.25, 1.09] |
|  | Lifetime Smoking | 0.04 | 0.43 | 0.09 | 632 | .927 | [-0.81, 0.88] |
|  | Currently Smoking | -0.73 | 0.72 | -1.02 | 632 | .310 | [-2.14, 0.68] |
|  | SD (Intercept) | 1.41 | 0.61 |  |  |  | [0.60, 3.31] |
|  | SD (Observations) | 4.40 | 0.12 |  |  |  | [4.17, 4.65] |
| AIC = 3796.13; AICc = 3796.71; BIC = 3854.23; R^2^ cond = 0.13; R^2^ marg = 0.04; ICC = 0.09; RMSE = 4.36; Sigma = 4.40 | | | | | | | |
| RV GLS | (Intercept) | -24.49 | 1.79 | -13.69 | 632 | < .001*** | [-28.01, -20.98] |
|  | Case | 0.10 | 0.30 | 0.33 | 632 | .742 | [-0.49, 0.69] |
|  | Age | 0.01 | 0.01 | 0.87 | 632 | .384 | [-0.01, 0.04] |
|  | Sex female | -1.56 | 0.33 | -4.76 | 632 | < .001*** | [-2.21, -0.92] |
|  | BMI | 0.02 | 0.03 | 0.48 | 632 | .634 | [-0.05, 0.08] |
|  | Diastolic BP | 0.03 | 0.02 | 1.15 | 632 | .249 | [-0.02, 0.07] |
|  | Systolic BP | -0.01 | 0.01 | -0.63 | 632 | .526 | [-0.04, 0.02] |
|  | HR | 0.00 | 0.01 | 0.35 | 632 | .726 | [-0.02, 0.03] |
|  | Cardiac Medication | 0.09 | 0.49 | 0.19 | 632 | .847 | [-0.87, 1.05] |
|  | Lifetime Smoking | 0.19 | 0.35 | 0.55 | 632 | .582 | [-0.50, 0.89] |
|  | Currently Smoking | -0.74 | 0.59 | -1.25 | 632 | .213 | [-1.89, 0.42] |
|  | SD (Intercept) | 1.19 | 0.52 |  |  |  | [0.51, 2.80] |
|  | SD (Observations) | 3.62 | 0.10 |  |  |  | [3.42, 3.82] |
| AIC = 3546.49; AICc = 3547.07; BIC = 3604.59; R^2^ cond = 0.14; R^2^ marg = 0.04; ICC = 0.10; RMSE = 3.58; Sigma = 3.62 | | | | | | | |
| RA area | (Intercept) | 14.62 | 1.30 | 11.23 | 616 | < .001*** | [12.07, 17.18] |
|  | Case | 0.25 | 0.21 | 1.15 | 616 | .252 | [-0.18, 0.67] |
|  | Age | 0.03 | 0.01 | 2.95 | 616 | .003** | [0.01, 0.04] |
|  | Sex female | -2.29 | 0.23 | -9.88 | 616 | < .001*** | [-2.74, -1.83] |
|  | BMI | 0.12 | 0.02 | 5.16 | 616 | < .001*** | [0.07, 0.16] |
|  | Diastolic BP | 0.00 | 0.02 | 0.12 | 616 | .907 | [-0.03, 0.03] |
|  | Systolic BP | -0.00 | 0.01 | -0.31 | 616 | .758 | [-0.02, 0.02] |
|  | HR | -0.05 | 0.01 | -5.79 | 616 | < .001*** | [-0.07, -0.04] |
|  | Cardiac Medication | -1.05 | 0.33 | -3.19 | 616 | .001** | [-1.70, -0.40] |
|  | Lifetime Smoking | -0.42 | 0.25 | -1.69 | 616 | .092 | [-0.91, 0.07] |
|  | Currently Smoking | -0.02 | 0.40 | -0.06 | 616 | .951 | [-0.81, 0.76] |
|  | SD (Intercept) | 1.03 | 0.44 |  |  |  | [0.44, 2.40] |
|  | SD (Observations) | 2.54 | 0.07 |  |  |  | [2.40, 2.69] |
| AIC = 3025.25; AICc = 3025.84; BIC = 3083.02; R^2^ cond = 0.33; R^2^ marg = 0.22; ICC = 0.14; RMSE = 2.51; Sigma = 2.54 | | | | | | | |
|  | | | | | | | |
| Note. Linear regression models (LMM) using case as predictor; controlled for age (per year), sex, BMI (per kg/m²), diastolic and systolic blood pressure (per mmHg) and heart rate (per bpm), smoking, center. Uncertainty intervals (equal-tailed) and p-values (two-tailed). * p < .05, ** p < .01, *** p < .001. Total: 930 participants: 512 post-COVID syndrome (PCS) and 418 recovered controls (CON).  Abbreviations: LV EF A4C = left ventricular ejection fraction in four-chamber view, BMI = body mass index, HR = heart rate, A2C = two-chamber view, EDVi A4C = end-diastolic volume in four-chamber view indexed to body surface area, ESVi = end-systolic volume indexed to body surface area, LV GLS = left ventricular global longitudinal strain, LA diam = left atrium diameter, RV = right ventricle, mid = midventricular, E/A = E/A ratio, RV = right ventricle, TAPSE = tricuspid annular plane systolic excursion, RV FWS = right ventricle free wall strain, RV GLS = right ventricle global longitudinal strain, RA = right atrium | | | | | | | |

**Supplemental Table 4 Pathologic and non-pathologic strain values between post-COVID syndrome and recovered controls**

|  | PCS | CON | p values |
| --- | --- | --- | --- |
| pathological LV GLS > -20% | 184 (44.4%) | 118 (36.3%) | 0.031* |
| normal LV GLS < -20% | 230 (55.6%) | 207 (63.7%) |  |
| pathological RV GLS > -21.5% | 149 (33%) | 111 (32.7%) | 1.000 |
| normal RV GLS < -21.5% | 303 (67%) | 228 (67.3%) |  |
| pathological RV FWS > -23.0% | 82 (18.1%) | 43 (12.7%) | 0.050 |
| normal RV GLS < -23.0% | 372 (81.9%) | 296 (87.3%) |  |
| Abbreviations: LV GLS = left ventricular global longitudinal strain, RV GLS = right ventricle global longitudinal strain, RV FWS = right ventricle free wall strain | | | |

**Supplemental Table 5 Differences in adjusted means between participants with post-COVID syndrome with certain symptoms**

|  | LV GLS, % | RV GLS, % | RV FWS, % | E/A | TAPSE |
| --- | --- | --- | --- | --- | --- |
| Wheezing | -20.40 (0.43) | -23.37 (0.87) | -27.42 (1.09) | 1.12 (0.05) | 24.18 (0.83) |
| No wheezing | -20.29 (0.39) | -22.75 (0.82) | -26.98 (1.03) | 1.14 (0.04) | 24.56 (0.78) |
| Dyspnea | -20.28 (0.38) | -23.06 (0.81) | -27.26 (1.02) | 1.13 (0.05) | 24.32 (0.78) |
| No dyspnea | -20.41 (0.42) | -22.41 (0.86) | -26.57 (1.08) | 1.14 (0.05) | 24.88 (0.83) |
| Chest pain | -20.23 (0.41) | -22.93 (0.85) | -27.13 (1.07) | 1.09 (0.05)* | 24.25 (0.81) |
| No chest pain | -20.35 (0.38) | -22.90 (0.81) | -27.09 (1.02) | 1.15 (0.05) | 24.53 (0.78) |
| CF | -20.31 (0.38) | -23.06 (0.81) | -27.10 (1.02) | 1.12 (0.05) | 24.43 (0.79) |
| No CF | -20.32 (0.42) | -22.76 (0.85) | -27.26 (1.07) | 1.13 (0.05) | 24.35 (0.83) |
| RPE | -20.23 (0.38)* | -22.85 (0.81) | -27.02 (1.01) | 1.13 (0.04) | 24.41 (0.78) |
| No RPE | -20.74 (0.43) | -23.16 (0.88) | -27.50 (1.10) | 1.15 (0.05) | 24.61 (0.85) |

Means and SE (standard error). Abbreviations: LV GLS = left ventricular global longitudinal strain, RV GLS = right ventricle global longitudinal strain, RV FWS = right ventricle free wall strain, E/A = E/A ratio, TAPSE = tricuspid annular plane systolic excursion, CF = chronic fatigue, RPE = rapid physical exhaustion

**Supplemental Table 6 Differences in echocardiographic parameters between participants with post-COVID syndrome with vs. without certain symptoms (adjusted)**

| Wheezing |  | b | SE | t | df | p | 95% CI |
| --- | --- | --- | --- | --- | --- | --- | --- |
| LV GLS | (Intercept) | -25.95 | 1.30 | -19.90 | 398 | < .001*** | [-28.52, -23.39] |
|  | Wheezing | -0.11 | 0.26 | -0.43 | 398 | .665 | [-0.63, 0.40] |
|  | Age | 0.02 | 0.01 | 2.40 | 398 | .017* | [0.00, 0.04] |
|  | Sex (female) | -0.77 | 0.22 | -3.46 | 398 | .001*** | [-1.21, -0.33] |
|  | BMI | 0.01 | 0.02 | 0.42 | 398 | .677 | [-0.03, 0.05] |
|  | Diastolic BP | 0.04 | 0.02 | 2.21 | 398 | .028* | [0.00, 0.07] |
|  | Systolic BP | 0.00 | 0.01 | 0.25 | 398 | .799 | [-0.02, 0.02] |
|  | HR | 0.02 | 0.01 | 2.37 | 398 | .018* | [0.00, 0.04] |
|  | Cardiac medication | -0.16 | 0.24 | -0.65 | 398 | .515 | [-0.62, 0.31] |
|  | Lifetime Smoking | 0.03 | 0.23 | 0.14 | 398 | .892 | [-0.43, 0.49] |
|  | Currently Smoking | -0.28 | 0.38 | -0.72 | 398 | .469 | [-1.02, 0.47] |
|  | SD (Intercept) | 0.69 | 0.31 |  |  |  | [0.29, 1.67] |
|  | SD (Observations) | 2.05 | 0.07 |  |  |  | [1.91, 2.20] |
| RV GLS | (Intercept) | -24.22 | 2.24 | -10.82 | 436 | < .001*** | [-28.62, -19.82] |
|  | Wheezing | -0.63 | 0.44 | -1.43 | 436 | .154 | [-1.49, 0.24] |
|  | Age | 0.00 | 0.02 | 0.21 | 436 | .835 | [-0.03, 0.03] |
|  | Sex (female) | -1.70 | 0.38 | -4.47 | 436 | < .001*** | [-2.44, -0.95] |
|  | BMI | 0.00 | 0.04 | 0.03 | 436 | .979 | [-0.07, 0.07] |
|  | Diastolic BP | 0.06 | 0.03 | 2.10 | 436 | .036* | [0.00, 0.11] |
|  | Systolic BP | -0.03 | 0.02 | -1.41 | 436 | .161 | [-0.06, 0.01] |
|  | HR | 0.01 | 0.02 | 0.42 | 436 | .674 | [-0.03, 0.04] |
|  | Cardiac medication | 0.33 | 0.41 | 0.82 | 436 | .414 | [-0.47, 1.14] |
|  | Lifetime Smoking | 0.48 | 0.40 | 1.20 | 436 | .231 | [-0.31, 1.27] |
|  | Currently Smoking | -0.28 | 0.63 | -0.44 | 436 | .661 | [-1.52, 0.96] |
|  | SD (Intercept) | 1.54 | 0.66 |  |  |  | [0.66, 3.58] |
|  | SD (Observations) | 3.64 | 0.12 |  |  |  | [3.40, 3.88] |
| RV FWS | (Intercept) | -28.80 | 2.70 | -10.66 | 438 | < .001*** | [-34.11, -23.50] |
|  | Wheezing | -0.44 | 0.52 | -0.84 | 438 | .404 | [-1.47, 0.59] |
|  | Age | -0.03 | 0.02 | -1.48 | 438 | .141 | [-0.06, 0.01] |
|  | Sex (female) | -1.92 | 0.45 | -4.22 | 438 | < .001*** | [-2.81, -1.02] |
|  | BMI | 0.03 | 0.04 | 0.62 | 438 | .533 | [-0.06, 0.11] |
|  | Diastolic BP | 0.03 | 0.03 | 1.06 | 438 | .291 | [-0.03, 0.10] |
|  | Systolic BP | -0.00 | 0.02 | -0.16 | 438 | .871 | [-0.05, 0.04] |
|  | HR | 0.01 | 0.02 | 0.65 | 438 | .515 | [-0.03, 0.05] |
|  | Cardiac medication | 0.33 | 0.49 | 0.67 | 438 | .500 | [-0.63, 1.29] |
|  | Lifetime Smoking | 0.60 | 0.48 | 1.24 | 438 | .216 | [-0.35, 1.54] |
|  | Currently Smoking | -0.62 | 0.76 | -0.81 | 438 | .416 | [-2.11, 0.87] |
|  | SD (Intercept) | 1.94 | 0.83 |  |  |  | [0.84, 4.49] |
|  | SD (Observations) | 4.37 | 0.15 |  |  |  | [4.09, 4.67] |
| E/A | (Intercept) | 2.81 | 0.15 | 18.77 | 440 | < .001*** | [2.51, 3.10] |
|  | Wheezing | -0.02 | 0.03 | -0.69 | 440 | .492 | [-0.08, 0.04] |
|  | Age | -0.01 | 0.00 | -12.54 | 440 | < .001*** | [-0.02, -0.01] |
|  | Sex (female) | -0.01 | 0.03 | -0.38 | 440 | .705 | [-0.06, 0.04] |
|  | BMI | -0.01 | 0.00 | -2.71 | 440 | .007** | [-0.01, -0.00] |
|  | Diastolic BP | -0.00 | 0.00 | -1.10 | 440 | .271 | [-0.01, 0.00] |
|  | Systolic BP | -0.00 | 0.00 | -1.42 | 440 | .157 | [-0.00, 0.00] |
|  | HR | -0.01 | 0.00 | -4.48 | 440 | < .001*** | [-0.01, -0.00] |
|  | Cardiac medication | -0.04 | 0.03 | -1.37 | 440 | .170 | [-0.10, 0.02] |
|  | Lifetime Smoking | 0.00 | 0.03 | 0.13 | 440 | .900 | [-0.05, 0.06] |
|  | Currently Smoking | -0.03 | 0.04 | -0.58 | 440 | .564 | [-0.11, 0.06] |
|  | SD (Intercept) | 0.08 | 0.04 |  |  |  | [0.03, 0.20] |
|  | SD (Observations) | 0.26 | 0.01 |  |  |  | [0.25, 0.28] |
| TAPSE | (Intercept) | 23.43 | 2.06 | 11.39 | 574 | < .001*** | [19.39, 27.47] |
|  | Wheezing | -0.38 | 0.40 | -0.94 | 574 | .345 | [-1.16, 0.41] |
|  | Age | 0.01 | 0.02 | 0.72 | 574 | .470 | [-0.02, 0.04] |
|  | Sex (female) | -0.08 | 0.36 | -0.23 | 574 | .821 | [-0.79, 0.63] |
|  | BMI | 0.14 | 0.03 | 4.40 | 574 | < .001*** | [0.08, 0.20] |
|  | Diastolic BP | -0.08 | 0.03 | -3.15 | 574 | .002** | [-0.13, -0.03] |
|  | Systolic BP | 0.05 | 0.02 | 2.76 | 574 | .006** | [0.01, 0.08] |
|  | HR | -0.03 | 0.02 | -2.21 | 574 | .028* | [-0.07, -0.00] |
|  | Cardiac medication | -0.55 | 0.38 | -1.43 | 574 | .154 | [-1.30, 0.21] |
|  | Lifetime Smoking | -0.13 | 0.37 | -0.35 | 574 | .726 | [-0.85, 0.60] |
|  | Currently Smoking | -0.21 | 0.63 | -0.34 | 574 | .735 | [-1.44, 1.02] |
|  | SD (Intercept) | 1.47 | 0.63 |  |  |  | [0.63, 3.42] |
|  | SD (Observations) | 3.92 | 0.12 |  |  |  | [3.70, 4.16] |
| Dyspnea |  | b | SE | t | df | p | 95% CI |
| LV GLS | (Intercept) | -25.97 | 1.30 | -19.91 | 398 | < .001*** | [-28.53, -23.40] |
|  | Dyspnea | 0.13 | 0.23 | 0.57 | 398 | .572 | [-0.33, 0.59] |
|  | Age | 0.02 | 0.01 | 2.47 | 398 | .014* | [0.00, 0.04] |
|  | Sex (female) | -0.78 | 0.22 | -3.48 | 398 | .001*** | [-1.22, -0.34] |
|  | BMI | 0.01 | 0.02 | 0.24 | 398 | .812 | [-0.04, 0.05] |
|  | Diastolic BP | 0.04 | 0.02 | 2.13 | 398 | .034* | [0.00, 0.07] |
|  | Systolic BP | 0.00 | 0.01 | 0.31 | 398 | .756 | [-0.02, 0.02] |
|  | HR | 0.02 | 0.01 | 2.37 | 398 | .018* | [0.00, 0.04] |
|  | Cardiac medication | -0.16 | 0.24 | -0.67 | 398 | .505 | [-0.63, 0.31] |
|  | Lifetime Smoking | 0.01 | 0.23 | 0.06 | 398 | .949 | [-0.44, 0.47] |
|  | Currently Smoking | -0.31 | 0.38 | -0.82 | 398 | .412 | [-1.05, 0.43] |
|  | SD (Intercept) | 0.69 | 0.31 |  |  |  | [0.29, 1.67] |
|  | SD (Observations) | 2.05 | 0.07 |  |  |  | [1.91, 2.20] |
| RV GLS | (Intercept) | -24.03 | 2.24 | -10.74 | 436 | < .001*** | [-28.42, -19.63] |
|  | Dyspnea | -0.65 | 0.41 | -1.59 | 436 | .112 | [-1.45, 0.15] |
|  | Age | 0.00 | 0.02 | 0.21 | 436 | .833 | [-0.03, 0.03] |
|  | Sex (female) | -1.64 | 0.38 | -4.31 | 436 | < .001*** | [-2.39, -0.89] |
|  | BMI | -0.00 | 0.03 | -0.08 | 436 | .934 | [-0.07, 0.07] |
|  | Diastolic BP | 0.06 | 0.03 | 2.22 | 436 | .027* | [0.01, 0.12] |
|  | Systolic BP | -0.03 | 0.02 | -1.51 | 436 | .132 | [-0.06, 0.01] |
|  | HR | 0.01 | 0.02 | 0.59 | 436 | .554 | [-0.02, 0.04] |
|  | Cardiac medication | 0.34 | 0.41 | 0.83 | 436 | .404 | [-0.46, 1.14] |
|  | Lifetime Smoking | 0.48 | 0.40 | 1.19 | 436 | .234 | [-0.31, 1.27] |
|  | Currently Smoking | -0.34 | 0.63 | -0.54 | 436 | .592 | [-1.57, 0.90] |
|  | SD (Intercept) | 1.52 | 0.66 |  |  |  | [0.65, 3.54] |
|  | SD (Observations) | 3.63 | 0.12 |  |  |  | [3.40, 3.88] |
| RV FWS | (Intercept) | -28.60 | 2.70 | -10.60 | 438 | < .001*** | [-33.90, -23.29] |
|  | Dyspnea | -0.69 | 0.49 | -1.40 | 438 | .161 | [-1.65, 0.28] |
|  | Age | -0.03 | 0.02 | -1.49 | 438 | .138 | [-0.07, 0.01] |
|  | Sex (female) | -1.86 | 0.46 | -4.08 | 438 | < .001*** | [-2.76, -0.96] |
|  | BMI | 0.03 | 0.04 | 0.64 | 438 | .522 | [-0.05, 0.11] |
|  | Diastolic BP | 0.04 | 0.03 | 1.17 | 438 | .243 | [-0.03, 0.10] |
|  | Systolic BP | -0.01 | 0.02 | -0.28 | 438 | .783 | [-0.05, 0.04] |
|  | HR | 0.02 | 0.02 | 0.78 | 438 | .437 | [-0.02, 0.06] |
|  | Cardiac medication | 0.34 | 0.49 | 0.69 | 438 | .488 | [-0.62, 1.30] |
|  | Lifetime Smoking | 0.61 | 0.48 | 1.28 | 438 | .201 | [-0.33, 1.56] |
|  | Currently Smoking | -0.64 | 0.75 | -0.85 | 438 | .397 | [-2.12, 0.84] |
|  | SD (Intercept) | 1.92 | 0.83 |  |  |  | [0.83, 4.46] |
|  | SD (Observations) | 4.36 | 0.15 |  |  |  | [4.08, 4.66] |
| E/A | (Intercept) | 2.81 | 0.15 | 18.67 | 440 | < .001*** | [2.52, 3.11] |
|  | Dyspnea | -0.01 | 0.03 | -0.45 | 440 | .653 | [-0.07, 0.05] |
|  | Age | -0.01 | 0.00 | -12.54 | 440 | < .001*** | [-0.02, -0.01] |
|  | Sex (female) | -0.01 | 0.03 | -0.37 | 440 | .710 | [-0.06, 0.04] |
|  | BMI | -0.01 | 0.00 | -2.77 | 440 | .006** | [-0.01, -0.00] |
|  | Diastolic BP | -0.00 | 0.00 | -1.02 | 440 | .308 | [-0.01, 0.00] |
|  | Systolic BP | -0.00 | 0.00 | -1.46 | 440 | .145 | [-0.00, 0.00] |
|  | HR | -0.01 | 0.00 | -4.47 | 440 | < .001*** | [-0.01, -0.00] |
|  | Cardiac medication | -0.04 | 0.03 | -1.38 | 440 | .170 | [-0.10, 0.02] |
|  | Lifetime Smoking | 0.00 | 0.03 | 0.09 | 440 | .930 | [-0.05, 0.06] |
|  | Currently Smoking | -0.03 | 0.04 | -0.65 | 440 | .518 | [-0.12, 0.06] |
|  | SD (Intercept) | 0.08 | 0.04 |  |  |  | [0.03, 0.20] |
|  | SD (Observations) | 0.26 | 0.01 |  |  |  | [0.25, 0.28] |
| TAPSE | (Intercept) | 23.65 | 2.06 | 11.46 | 574 | < .001*** | [19.60, 27.71] |
|  | Dyspnea | -0.56 | 0.39 | -1.44 | 574 | .151 | [-1.31, 0.20] |
|  | Age | 0.01 | 0.02 | 0.65 | 574 | .514 | [-0.02, 0.04] |
|  | Sex (female) | -0.05 | 0.36 | -0.15 | 574 | .884 | [-0.76, 0.66] |
|  | BMI | 0.14 | 0.03 | 4.48 | 574 | < .001*** | [0.08, 0.20] |
|  | Diastolic BP | -0.08 | 0.03 | -3.00 | 574 | .003** | [-0.13, -0.03] |
|  | Systolic BP | 0.04 | 0.02 | 2.64 | 574 | .008** | [0.01, 0.08] |
|  | HR | -0.03 | 0.02 | -2.16 | 574 | .031* | [-0.06, -0.00] |
|  | Cardiac medication | -0.55 | 0.38 | -1.45 | 574 | .149 | [-1.30, 0.20] |
|  | Lifetime Smoking | -0.12 | 0.37 | -0.32 | 574 | .750 | [-0.84, 0.61] |
|  | Currently Smoking | -0.23 | 0.62 | -0.38 | 574 | .706 | [-1.45, 0.98] |
|  | SD (Intercept) | 1.48 | 0.64 |  |  |  | [0.63, 3.43] |
|  | SD (Observations) | 3.92 | 0.12 |  |  |  | [3.70, 4.15] |
| Chest Pain |  | b | SE | t | df | p | 95% CI |
| LV GLS | (Intercept) | -25.93 | 1.30 | -19.88 | 398 | < .001*** | [-28.50, -23.37] |
|  | Chest Pain | 0.12 | 0.24 | 0.51 | 398 | .607 | [-0.34, 0.59] |
|  | Age | 0.02 | 0.01 | 2.43 | 398 | .016* | [0.00, 0.04] |
|  | Sex (female) | -0.77 | 0.22 | -3.44 | 398 | .001*** | [-1.20, -0.33] |
|  | BMI | 0.01 | 0.02 | 0.29 | 398 | .771 | [-0.04, 0.05] |
|  | Diastolic BP | 0.04 | 0.02 | 2.22 | 398 | .027* | [0.00, 0.07] |
|  | Systolic BP | 0.00 | 0.01 | 0.25 | 398 | .800 | [-0.02, 0.02] |
|  | HR | 0.02 | 0.01 | 2.37 | 398 | .018* | [0.00, 0.04] |
|  | Cardiac medication | -0.16 | 0.24 | -0.68 | 398 | .497 | [-0.63, 0.31] |
|  | Lifetime Smoking | 0.01 | 0.23 | 0.03 | 398 | .974 | [-0.45, 0.47] |
|  | Currently Smoking | -0.31 | 0.38 | -0.82 | 398 | .414 | [-1.05, 0.43] |
|  | SD (Intercept) | 0.69 | 0.31 |  |  |  | [0.29, 1.67] |
|  | SD (Observations) | 2.05 | 0.07 |  |  |  | [1.91, 2.20] |
| RV GLS | (Intercept) | -24.21 | 2.24 | -10.79 | 436 | < .001*** | [-28.62, -19.80] |
|  | Chest Pain | -0.03 | 0.40 | -0.07 | 436 | .946 | [-0.81, 0.75] |
|  | Age | 0.00 | 0.02 | 0.26 | 436 | .798 | [-0.03, 0.04] |
|  | Sex (female) | -1.71 | 0.38 | -4.49 | 436 | < .001*** | [-2.45, -0.96] |
|  | BMI | -0.01 | 0.03 | -0.29 | 436 | .771 | [-0.08, 0.06] |
|  | Diastolic BP | 0.06 | 0.03 | 2.06 | 436 | .040* | [0.00, 0.11] |
|  | Systolic BP | -0.02 | 0.02 | -1.34 | 436 | .180 | [-0.06, 0.01] |
|  | HR | 0.01 | 0.02 | 0.49 | 436 | .621 | [-0.03, 0.04] |
|  | Cardiac medication | 0.33 | 0.41 | 0.80 | 436 | .422 | [-0.48, 1.13] |
|  | Lifetime Smoking | 0.43 | 0.40 | 1.07 | 436 | .287 | [-0.36, 1.22] |
|  | Currently Smoking | -0.39 | 0.63 | -0.63 | 436 | .531 | [-1.63, 0.84] |
|  | SD (Intercept) | 1.52 | 0.66 |  |  |  | [0.65, 3.54] |
|  | SD (Observations) | 3.64 | 0.12 |  |  |  | [3.41, 3.89] |
| RV FWS | (Intercept) | -28.80 | 2.70 | -10.65 | 438 | < .001*** | [-34.11, -23.49] |
|  | Chest Pain | -0.04 | 0.47 | -0.08 | 438 | .937 | [-0.97, 0.90] |
|  | Age | -0.03 | 0.02 | -1.44 | 438 | .150 | [-0.06, 0.01] |
|  | Sex (female) | -1.92 | 0.45 | -4.23 | 438 | < .001*** | [-2.82, -1.03] |
|  | BMI | 0.02 | 0.04 | 0.46 | 438 | .648 | [-0.06, 0.10] |
|  | Diastolic BP | 0.03 | 0.03 | 1.03 | 438 | .302 | [-0.03, 0.10] |
|  | Systolic BP | -0.00 | 0.02 | -0.13 | 438 | .900 | [-0.05, 0.04] |
|  | HR | 0.01 | 0.02 | 0.70 | 438 | .487 | [-0.03, 0.05] |
|  | Cardiac medication | 0.33 | 0.49 | 0.66 | 438 | .507 | [-0.64, 1.29] |
|  | Lifetime Smoking | 0.56 | 0.48 | 1.16 | 438 | .245 | [-0.39, 1.51] |
|  | Currently Smoking | -0.70 | 0.75 | -0.93 | 438 | .355 | [-2.18, 0.78] |
|  | SD (Intercept) | 1.93 | 0.83 |  |  |  | [0.83, 4.46] |
|  | SD (Observations) | 4.37 | 0.15 |  |  |  | [4.09, 4.67] |
| E/A | (Intercept) | 2.82 | 0.15 | 18.86 | 440 | < .001*** | [2.52, 3.11] |
|  | Chest Pain | -0.06 | 0.03 | -2.06 | 440 | .040* | [-0.11, -0.00] |
|  | Age | -0.01 | 0.00 | -12.57 | 440 | < .001*** | [-0.02, -0.01] |
|  | Sex (female) | -0.01 | 0.03 | -0.38 | 440 | .701 | [-0.06, 0.04] |
|  | BMI | -0.01 | 0.00 | -2.92 | 440 | .004** | [-0.01, -0.00] |
|  | Diastolic BP | -0.00 | 0.00 | -1.15 | 440 | .252 | [-0.01, 0.00] |
|  | Systolic BP | -0.00 | 0.00 | -1.38 | 440 | .167 | [-0.00, 0.00] |
|  | HR | -0.01 | 0.00 | -4.41 | 440 | < .001*** | [-0.01, -0.00] |
|  | Cardiac medication | -0.04 | 0.03 | -1.31 | 440 | .191 | [-0.10, 0.02] |
|  | Lifetime Smoking | 0.01 | 0.03 | 0.29 | 440 | .770 | [-0.05, 0.06] |
|  | Currently Smoking | -0.02 | 0.04 | -0.56 | 440 | .578 | [-0.11, 0.06] |
|  | SD (Intercept) | 0.08 | 0.04 |  |  |  | [0.03, 0.20] |
|  | SD (Observations) | 0.26 | 0.01 |  |  |  | [0.25, 0.28] |
| TAPSE | (Intercept) | 23.46 | 2.06 | 11.41 | 574 | < .001*** | [19.42, 27.49] |
|  | Chest Pain | -0.28 | 0.38 | -0.75 | 574 | .456 | [-1.02, 0.46] |
|  | Age | 0.01 | 0.02 | 0.75 | 574 | .456 | [-0.02, 0.04] |
|  | Sex (female) | -0.08 | 0.36 | -0.22 | 574 | .826 | [-0.79, 0.63] |
|  | BMI | 0.13 | 0.03 | 4.29 | 574 | < .001*** | [0.07, 0.19] |
|  | Diastolic BP | -0.08 | 0.03 | -3.16 | 574 | .002** | [-0.13, -0.03] |
|  | Systolic BP | 0.05 | 0.02 | 2.79 | 574 | .005** | [0.01, 0.08] |
|  | HR | -0.03 | 0.02 | -2.13 | 574 | .033* | [-0.06, -0.00] |
|  | Cardiac medication | -0.55 | 0.38 | -1.43 | 574 | .153 | [-1.30, 0.20] |
|  | Lifetime Smoking | -0.13 | 0.37 | -0.35 | 574 | .727 | [-0.86, 0.60] |
|  | Currently Smoking | -0.28 | 0.62 | -0.45 | 574 | .654 | [-1.49, 0.94] |
|  | SD (Intercept) | 1.46 | 0.63 |  |  |  | [0.62, 3.39] |
|  | SD (Observations) | 3.92 | 0.12 |  |  |  | [3.70, 4.16] |
| CF |  | b | SE | t | df | p | 95% CI |
| LV GLS | (Intercept) | -25.93 | 1.33 | -19.56 | 397 | < .001*** | [-28.54, -23.33] |
|  | CF | 0.01 | 0.23 | 0.05 | 397 | .960 | [-0.43, 0.46] |
|  | Age | 0.02 | 0.01 | 2.43 | 397 | .016* | [0.00, 0.04] |
|  | Sex (female) | -0.77 | 0.23 | -3.43 | 397 | .001*** | [-1.22, -0.33] |
|  | BMI | 0.01 | 0.02 | 0.31 | 397 | .757 | [-0.04, 0.05] |
|  | Diastolic BP | 0.04 | 0.02 | 2.20 | 397 | .028* | [0.00, 0.07] |
|  | Systolic BP | 0.00 | 0.01 | 0.25 | 397 | .800 | [-0.02, 0.02] |
|  | HR | 0.02 | 0.01 | 2.38 | 397 | .018* | [0.00, 0.04] |
|  | Cardiac medication | -0.15 | 0.24 | -0.64 | 397 | .521 | [-0.62, 0.32] |
|  | Lifetime Smoking | 0.02 | 0.23 | 0.10 | 397 | .918 | [-0.43, 0.48] |
|  | Currently Smoking | -0.29 | 0.38 | -0.75 | 397 | .454 | [-1.04, 0.47] |
|  | SD (Intercept) | 0.69 | 0.31 |  |  |  | [0.29, 1.67] |
|  | SD (Observations) | 2.05 | 0.07 |  |  |  | [1.91, 2.20] |
| RV GLS | (Intercept) | -24.29 | 2.28 | -10.67 | 435 | < .001*** | [-28.76, -19.82] |
|  | CF | -0.30 | 0.38 | -0.79 | 435 | .429 | [-1.06, 0.45] |
|  | Age | 0.00 | 0.02 | 0.14 | 435 | .889 | [-0.03, 0.03] |
|  | Sex (female) | -1.65 | 0.38 | -4.34 | 435 | < .001*** | [-2.40, -0.90] |
|  | BMI | -0.01 | 0.03 | -0.24 | 435 | .808 | [-0.08, 0.06] |
|  | Diastolic BP | 0.06 | 0.03 | 2.03 | 435 | .043* | [0.00, 0.11] |
|  | Systolic BP | -0.02 | 0.02 | -1.20 | 435 | .231 | [-0.06, 0.01] |
|  | HR | 0.01 | 0.02 | 0.56 | 435 | .575 | [-0.02, 0.04] |
|  | Cardiac medication | 0.28 | 0.41 | 0.69 | 435 | .491 | [-0.52, 1.09] |
|  | Lifetime Smoking | 0.44 | 0.40 | 1.11 | 435 | .267 | [-0.34, 1.23] |
|  | Currently Smoking | -0.53 | 0.64 | -0.83 | 435 | .406 | [-1.78, 0.72] |
|  | SD (Intercept) | 1.52 | 0.66 |  |  |  | [0.65, 3.54] |
|  | SD (Observations) | 3.63 | 0.12 |  |  |  | [3.40, 3.88] |
| RV FWS | (Intercept) | -29.16 | 2.75 | -10.60 | 437 | < .001*** | [-34.56, -23.75] |
|  | CF | 0.16 | 0.46 | 0.34 | 437 | .733 | [-0.75, 1.06] |
|  | Age | -0.03 | 0.02 | -1.43 | 437 | .153 | [-0.06, 0.01] |
|  | Sex (female) | -1.88 | 0.46 | -4.11 | 437 | < .001*** | [-2.78, -0.98] |
|  | BMI | 0.02 | 0.04 | 0.44 | 437 | .663 | [-0.06, 0.10] |
|  | Diastolic BP | 0.03 | 0.03 | 1.00 | 437 | .316 | [-0.03, 0.10] |
|  | Systolic BP | -0.00 | 0.02 | -0.02 | 437 | .986 | [-0.04, 0.04] |
|  | HR | 0.01 | 0.02 | 0.71 | 437 | .480 | [-0.03, 0.05] |
|  | Cardiac medication | 0.29 | 0.49 | 0.60 | 437 | .550 | [-0.67, 1.26] |
|  | Lifetime Smoking | 0.56 | 0.48 | 1.17 | 437 | .242 | [-0.38, 1.50] |
|  | Currently Smoking | -0.82 | 0.77 | -1.07 | 437 | .285 | [-2.32, 0.69] |
|  | SD (Intercept) | 1.94 | 0.83 |  |  |  | [0.83, 4.49] |
|  | SD (Observations) | 4.37 | 0.15 |  |  |  | [4.09, 4.67] |
| E/A | (Intercept) | 2.79 | 0.15 | 18.34 | 439 | < .001*** | [2.49, 3.09] |
|  | CF | -0.01 | 0.03 | -0.40 | 439 | .692 | [-0.06, 0.04] |
|  | Age | -0.01 | 0.00 | -12.60 | 439 | < .001*** | [-0.02, -0.01] |
|  | Sex (female) | -0.01 | 0.03 | -0.23 | 439 | .822 | [-0.06, 0.05] |
|  | BMI | -0.01 | 0.00 | -2.88 | 439 | .004** | [-0.01, -0.00] |
|  | Diastolic BP | -0.00 | 0.00 | -1.13 | 439 | .260 | [-0.01, 0.00] |
|  | Systolic BP | -0.00 | 0.00 | -1.26 | 439 | .207 | [-0.00, 0.00] |
|  | HR | -0.01 | 0.00 | -4.38 | 439 | < .001*** | [-0.01, -0.00] |
|  | Cardiac medication | -0.04 | 0.03 | -1.50 | 439 | .134 | [-0.10, 0.01] |
|  | Lifetime Smoking | 0.00 | 0.03 | 0.10 | 439 | .924 | [-0.05, 0.06] |
|  | Currently Smoking | -0.04 | 0.04 | -0.87 | 439 | .387 | [-0.13, 0.05] |
|  | SD (Intercept) | 0.08 | 0.04 |  |  |  | [0.03, 0.20] |
|  | SD (Observations) | 0.26 | 0.01 |  |  |  | [0.25, 0.28] |
| TAPSE | (Intercept) | 23.18 | 2.09 | 11.09 | 573 | < .001*** | [19.08, 27.28] |
|  | CF | 0.08 | 0.36 | 0.23 | 573 | .816 | [-0.62, 0.79] |
|  | Age | 0.01 | 0.02 | 0.73 | 573 | .466 | [-0.02, 0.04] |
|  | Sex (female) | -0.05 | 0.36 | -0.13 | 573 | .900 | [-0.76, 0.67] |
|  | BMI | 0.13 | 0.03 | 4.27 | 573 | < .001*** | [0.07, 0.19] |
|  | Diastolic BP | -0.08 | 0.03 | -3.15 | 573 | .002** | [-0.13, -0.03] |
|  | Systolic BP | 0.05 | 0.02 | 2.85 | 573 | .005** | [0.01, 0.08] |
|  | HR | -0.03 | 0.02 | -2.15 | 573 | .032* | [-0.06, -0.00] |
|  | Cardiac medication | -0.59 | 0.38 | -1.52 | 573 | .128 | [-1.34, 0.17] |
|  | Lifetime Smoking | -0.16 | 0.37 | -0.42 | 573 | .672 | [-0.88, 0.57] |
|  | Currently Smoking | -0.39 | 0.63 | -0.62 | 573 | .537 | [-1.62, 0.84] |
|  | SD (Intercept) | 1.48 | 0.64 |  |  |  | [0.64, 3.44] |
|  | SD (Observations) | 3.92 | 0.12 |  |  |  | [3.70, 4.16] |
| RPE |  | b | SE | t | df | p | 95% CI |
| LV GLS | (Intercept) | -26.16 | 1.30 | -20.08 | 398 | < .001*** | [-28.72, -23.60] |
|  | RPE | 0.51 | 0.26 | 1.97 | 398 | .050* | [0.00, 1.02] |
|  | Age | 0.02 | 0.01 | 2.53 | 398 | .012* | [0.01, 0.04] |
|  | Sex (female) | -0.79 | 0.22 | -3.54 | 398 | < .001*** | [-1.22, -0.35] |
|  | BMI | 0.01 | 0.02 | 0.27 | 398 | .785 | [-0.04, 0.05] |
|  | Diastolic BP | 0.03 | 0.02 | 2.04 | 398 | .042* | [0.00, 0.07] |
|  | Systolic BP | 0.00 | 0.01 | 0.39 | 398 | .693 | [-0.02, 0.03] |
|  | HR | 0.02 | 0.01 | 2.21 | 398 | .028* | [0.00, 0.04] |
|  | Cardiac medication | -0.19 | 0.24 | -0.81 | 398 | .418 | [-0.66, 0.27] |
|  | Lifetime Smoking | -0.00 | 0.23 | -0.02 | 398 | .988 | [-0.46, 0.45] |
|  | Currently Smoking | -0.32 | 0.37 | -0.86 | 398 | .392 | [-1.06, 0.42] |
|  | SD (Intercept) | 0.69 | 0.31 |  |  |  | [0.29, 1.66] |
|  | SD (Observations) | 2.04 | 0.07 |  |  |  | [1.90, 2.19] |
| RV GLS | (Intercept) | -24.38 | 2.25 | -10.82 | 436 | < .001*** | [-28.80, -19.95] |
|  | RPE | 0.31 | 0.45 | 0.69 | 436 | .492 | [-0.57, 1.18] |
|  | Age | 0.00 | 0.02 | 0.27 | 436 | .788 | [-0.03, 0.04] |
|  | Sex (female) | -1.72 | 0.38 | -4.52 | 436 | < .001*** | [-2.46, -0.97] |
|  | BMI | -0.01 | 0.03 | -0.30 | 436 | .764 | [-0.08, 0.06] |
|  | Diastolic BP | 0.06 | 0.03 | 1.99 | 436 | .047* | [0.00, 0.11] |
|  | Systolic BP | -0.02 | 0.02 | -1.28 | 436 | .200 | [-0.06, 0.01] |
|  | HR | 0.01 | 0.02 | 0.44 | 436 | .661 | [-0.03, 0.04] |
|  | Cardiac medication | 0.33 | 0.41 | 0.80 | 436 | .425 | [-0.48, 1.13] |
|  | Lifetime Smoking | 0.41 | 0.40 | 1.01 | 436 | .313 | [-0.38, 1.20] |
|  | Currently Smoking | -0.42 | 0.63 | -0.66 | 436 | .507 | [-1.65, 0.82] |
|  | SD (Intercept) | 1.52 | 0.66 |  |  |  | [0.65, 3.54] |
|  | SD (Observations) | 3.64 | 0.12 |  |  |  | [3.41, 3.89] |
| RV FWS | (Intercept) | -29.07 | 2.72 | -10.71 | 438 | < .001*** | [-34.41, -23.73] |
|  | RPE | 0.48 | 0.53 | 0.90 | 438 | .367 | [-0.57, 1.53] |
|  | Age | -0.03 | 0.02 | -1.43 | 438 | .155 | [-0.06, 0.01] |
|  | Sex (female) | -1.94 | 0.45 | -4.27 | 438 | < .001*** | [-2.83, -1.05] |
|  | BMI | 0.02 | 0.04 | 0.44 | 438 | .659 | [-0.06, 0.10] |
|  | Diastolic BP | 0.03 | 0.03 | 0.95 | 438 | .342 | [-0.03, 0.10] |
|  | Systolic BP | -0.00 | 0.02 | -0.05 | 438 | .959 | [-0.04, 0.04] |
|  | HR | 0.01 | 0.02 | 0.63 | 438 | .531 | [-0.03, 0.05] |
|  | Cardiac medication | 0.32 | 0.49 | 0.65 | 438 | .513 | [-0.64, 1.28] |
|  | Lifetime Smoking | 0.52 | 0.48 | 1.09 | 438 | .276 | [-0.42, 1.47] |
|  | Currently Smoking | -0.73 | 0.75 | -0.97 | 438 | .331 | [-2.21, 0.75] |
|  | SD (Intercept) | 1.92 | 0.82 |  |  |  | [0.83, 4.45] |
|  | SD (Observations) | 4.37 | 0.15 |  |  |  | [4.09, 4.67] |
| E/A | (Intercept) | 2.82 | 0.15 | 18.71 | 440 | < .001*** | [2.52, 3.11] |
|  | RPE | -0.02 | 0.03 | -0.69 | 440 | .490 | [-0.09, 0.04] |
|  | Age | -0.01 | 0.00 | -12.56 | 440 | < .001*** | [-0.02, -0.01] |
|  | Sex (female) | -0.01 | 0.03 | -0.36 | 440 | .716 | [-0.06, 0.04] |
|  | BMI | -0.01 | 0.00 | -2.88 | 440 | .004** | [-0.01, -0.00] |
|  | Diastolic BP | -0.00 | 0.00 | -1.05 | 440 | .296 | [-0.01, 0.00] |
|  | Systolic BP | -0.00 | 0.00 | -1.42 | 440 | .156 | [-0.00, 0.00] |
|  | HR | -0.01 | 0.00 | -4.39 | 440 | < .001*** | [-0.01, -0.00] |
|  | Cardiac medication | -0.04 | 0.03 | -1.35 | 440 | .178 | [-0.10, 0.02] |
|  | Lifetime Smoking | 0.00 | 0.03 | 0.11 | 440 | .916 | [-0.05, 0.06] |
|  | Currently Smoking | -0.03 | 0.04 | -0.66 | 440 | .510 | [-0.12, 0.06] |
|  | SD (Intercept) | 0.08 | 0.04 |  |  |  | [0.03, 0.20] |
|  | SD (Observations) | 0.26 | 0.01 |  |  |  | [0.25, 0.28] |
| TAPSE | (Intercept) | 23.51 | 2.07 | 11.34 | 574 | < .001*** | [19.44, 27.58] |
|  | RPE | -0.19 | 0.43 | -0.45 | 574 | .652 | [-1.04, 0.65] |
|  | Age | 0.01 | 0.02 | 0.73 | 574 | .468 | [-0.02, 0.04] |
|  | Sex (female) | -0.07 | 0.36 | -0.20 | 574 | .843 | [-0.78, 0.64] |
|  | BMI | 0.13 | 0.03 | 4.31 | 574 | < .001*** | [0.07, 0.19] |
|  | Diastolic BP | -0.08 | 0.03 | -3.11 | 574 | .002** | [-0.13, -0.03] |
|  | Systolic BP | 0.05 | 0.02 | 2.75 | 574 | .006** | [0.01, 0.08] |
|  | HR | -0.03 | 0.02 | -2.11 | 574 | .035* | [-0.06, -0.00] |
|  | Cardiac medication | -0.55 | 0.38 | -1.43 | 574 | .154 | [-1.30, 0.21] |
|  | Lifetime Smoking | -0.15 | 0.37 | -0.41 | 574 | .682 | [-0.88, 0.57] |
|  | Currently Smoking | -0.29 | 0.62 | -0.46 | 574 | .643 | [-1.50, 0.93] |
|  | SD (Intercept) | 1.47 | 0.63 |  |  |  | [0.63, 3.41] |
|  | SD (Observations) | 3.92 | 0.12 |  |  |  | [3.70, 4.16] |
| Note. Linear regression models (LMM) using specific symptoms (yes vs no) as Predictor; for age (per year), Sex, BMI, Diastolic and Systolic BP (per mmHg) and HR (per bpm), Smoking, Center, Uncertainty intervals (equal-tailed) and p-values (two-tailed), * p < .05, ** p controlled < .01, *** p < .001. Abbreviations: LV GLS = left ventricular global longitudinal strain, BMI = body mass index, BP = blood pressure, HR= heart rate, RV GLS = right ventricle global longitudinal strain, RV FWS = right ventricle free wall strain, E/A = E/A ratio, TAPSE = tricuspid annular plane systolic excursion, CF = chronic fatigue, RPE = rapid physical exhaustion | | | | | | | |

**Supplemental Table 7 Association of relative VO_2_max with echocardiographic parameters**

| Variable | b | SE | t | df | p | 95% CI |
| --- | --- | --- | --- | --- | --- | --- |
| (Intercept) | 20.45 | 3.06 | 6.68 | 962 | 0.00 | [14.44, 26.45] |
| LV EF A4C | 0.13 | 0.05 | 2.65 | 962 | 0.008** | [0.03, 0.22] |
| SD (Intercept) | 2.17 | 0.94 | - | - | - |  |
| SD (Observations) | 7.79 | 0.18 | - | - | - |  |
| (Intercept) | 28.64 | 3.25 | 8.82 | 734 | 0.00 | [22.26, 35.01] |
| LV EF A2C | -0.01 | 0.05 | -0.25 | 734 | 0.802 | [-0.11, 0.08] |
| SD (Intercept) | 2.16 | 0.97 | - | - | - |  |
| SD (Observations) | 7.83 | 0.20 | - | - | - |  |
| (Intercept) | 16.11 | 1.62 | 9.93 | 738 | 0.00 | [12.93, 19.30] |
| LV EDVi A4C | 0.24 | 0.03 | 9.05 | 738 | <0.001*** | [0.19, 0.29] |
| SD (Intercept) | 1.85 | 0.85 | - | - | - |  |
| SD (Observations) | 7.45 | 0.19 | - | - | - |  |
| (Intercept) | 15.42 | 1.42 | 10.87 | 727 | 0.00 | [12.64, 18.21] |
| LV EDVi A2C | 0.27 | 0.02 | 10.70 | 727 | <0.001*** | [0.22, 0.32] |
| SD (Intercept) | 1.49 | 0.72 | - | - | - |  |
| SD (Observations) | 7.30 | 0.19 | - | - | - |  |
| (Intercept) | 21.61 | 1.43 | 15.15 | 738 | 0.00 | [18.81, 24.41] |
| LV ESVi A4C | 0.32 | 0.05 | 5.91 | 738 | <0.001*** | [0.21, 0.42] |
| SD (Intercept) | 1.81 | 0.84 | - | - | - |  |
| SD (Observations) | 7.68 | 0.20 | - | - | - |  |
| (Intercept) | 19.47 | 1.21 | 16.14 | 727 | 0.00 | [17.10, 21.84] |
| LV ESVi A2C | 0.47 | 0.05 | 8.77 | 727 | <0.001*** | [0.36, 0.57] |
| SD (Intercept) | 1.31 | 0.66 | - | - | - |  |
| SD (Observations) | 7.47 | 0.20 | - | - | - |  |
| (Intercept) | 20.97 | 2.84 | 7.38 | 712 | 0.00 | [15.39, 26.55] |
| LV GLS | -0.38 | 0.13 | -2.93 | 712 | 0.004** | [-0.63, -0.13] |
| SD (Intercept) | 1.84 | 0.83 | - | - | - |  |
| SD (Observations) | 7.83 | 0.21 | - | - | - |  |
| (Intercept) | 29.78 | 2.08 | 14.32 | 978 | 0.00 | [25.70, 33.86] |
| LA diam | -0.06 | 0.05 | -1.09 | 978 | 0.277 | [-0.16, 0.05] |
| SD (Intercept) | 2.02 | 0.88 | - | - | - |  |
| SD (Observations) | 7.86 | 0.18 | - | - | - |  |
| (Intercept) | 26.51 | 1.72 | 15.42 | 756 | 0.00 | [23.13, 29.88] |
| LA area | 0.08 | 0.09 | 0.89 | 756 | 0.373 | [0.85, 5.00] |
| SD (Intercept) | 2.07 | 0.93 | - | - | - |  |
| SD (Observations) | 7.88 | 0.20 | - | - | - |  |
| (Intercept) | 17.98 | 1.25 | 14.38 | 758 | 0.00 | [15.52, 20.43] |
| E/A | 8.22 | 0.72 | 11.46 | 758 | <0.001*** | [0.70, 4.25] |
| SD (Intercept) | 1.73 | 0.79 | - | - | - |  |
| SD (Observations) | 7.28 | 0.19 | - | - | - |  |
| (Intercept) | 34.24 | 1.30 | 26.37 | 756 | 0.00 | [31.69, 36.79] |
| E/E’l | -1.03 | 0.14 | -7.25 | 756 | <0.001*** | [0.71, 4.35] |
| SD (Intercept) | 1.76 | 0.81 | - | - | - |  |
| SD (Observations) | 7.64 | 0.20 | - | - | - |  |
| (Intercept) | 35.97 | 1.34 | 26.89 | 758 | 0.00 | [33.35, 38.60] |
| E/E’m | -1.06 | 0.14 | -7.84 | 758 | <0.001*** | [0.59, 3.87] |
| SD (Intercept) | 1.51 | 0.73 | - | - | - |  |
| SD (Observations) | 7.62 | 0.20 | - | - | - |  |
| (Intercept) | 16.51 | 2.35 | 7.03 | 746 | 0.00 | [11.90, 21.12] |
| RV basal | 0.36 | 0.06 | 5.65 | 746 | <0.001*** | [1.02, 5.71] |
| SD (Intercept) | 2.42 | 1.06 | - | - | - |  |
| SD (Observations) | 7.74 | 0.20 | - | - | - |  |
| (Intercept) | 20.30 | 1.86 | 10.94 | 614 | 0.00 | [16.65, 23.94] |
| RV mid | 0.32 | 0.07 | 4.55 | 614 | <0.001*** | [0.73, 4.94] |
| SD (Intercept) | 1.90 | 0.93 | - | - | - |  |
| SD (Observations) | 7.63 | 0.22 | - | - | - |  |
| (Intercept) | 25.99 | 1.75 | 14.86 | 613 | 0.00 | [22.55, 29.42] |
| RV length | 0.03 | 0.03 | 1.08 | 613 | 0.282 | [0.87, 6.37] |
| SD (Intercept) | 2.35 | 1.20 | - | - | - |  |
| SD (Observations) | 7.75 | 0.22 | - | - | - |  |
| (Intercept) | 26.10 | 1.94 | 13.46 | 971 | 0.00 | [22.30, 29.91] |
| TAPSE | 0.07 | 0.06 | 1.12 | 971 | 0.262 | [0.90, 4.95] |
| SD (Intercept) | 2.12 | 0.92 | - | - | - |  |
| SD (Observations) | 7.86 | 0.18 | - | - | - |  |
| (Intercept) | 28.01 | 2.01 | 13.93 | 763 | 0.00 | [24.06, 31.95] |
| RV FWS | -0.02 | 0.06 | -0.33 | 763 | 0.744 | [0.78, 4.55] |
| SD (Intercept) | 1.89 | 0.85 | - | - | - |  |
| SD (Observations) | 7.95 | 0.20 | - | - | - |  |
| (Intercept) | 29.39 | 2.07 | 14.23 | 761 | 0.00 | [25.34, 33.45] |
| RV GLS | 0.03 | 0.08 | 0.44 | 761 | 0.663 | [0.79, 4.57] |
| SD (Intercept) | 1.90 | 0.85 | - | - | - |  |
| SD (Observations) | 7.94 | 0.20 | - | - | - |  |
| (Intercept) | 22.36 | 1.62 | 13.80 | 748 | 0.00 | [19.18, 25.54] |
| RA area | 0.40 | 0.10 | 4.09 | 748 | <0.001*** | [0.73, 4.48] |
| SD (Intercept) | 1.81 | 0.84 | - | - | - |  |
| SD (Observations) | 7.82 | 0.20 | - | - | - |  |

Note. Linear regression models (LMM) using echocardiographic parameters as predictors (and random intercept (center)) of VO_2_max. Uncertainty intervals (equal-tailed) and p-values (two-tailed), * p < .05, ** p < .01, *** p < 0.001.

Abbreviations: LV EF A4C = left ventricular ejection fraction in four-chamber view, A2C = two-chamber view, EDVi A4C = end-diastolic volume in four-chamber view indexed to body surface area, ESVi = end-systolic volume indexed to body surface area, LV GLS = left ventricular global longitudinal strain, LA diam = left atrium diameter, RV = right ventricle, mid = midventricular, E/A = E/A ratio, RV = right ventricle, TAPSE = tricuspid annular plane systolic excursion, RV FWS = right ventricle free wall strain, RV GLS = right ventricle global longitudinal strain, RA = right atrium
